# Resolving the heterogeneous tumour microenvironment in cardiac myxoma through single‐cell and spatial transcriptomics

**DOI:** 10.1002/ctm2.1581

**Published:** 2024-02-06

**Authors:** Xuanyu Liu, Huayan Shen, Jinxing Yu, Fengming Luo, Tianjiao Li, Qi Li, Xin Yuan, Yang Sun, Zhou Zhou

**Affiliations:** ^1^ State Key Laboratory of Cardiovascular Disease Fuwai Hospital National Center for Cardiovascular Diseases Chinese Academy of Medical Sciences and Peking Union Medical College Beijing China; ^2^ Beijing Key Laboratory for Molecular Diagnostics of Cardiovascular Diseases Center of Laboratory Medicine Fuwai Hospital Beijing China; ^3^ Department of Cardiovascular Surgery Fuwai Hospital Beijing China; ^4^ Department of Pathology Fuwai Hospital Beijing China

**Keywords:** cardiac myxoma, myxoma tumour cell, single‐cell RNA sequencing, spatial transcriptomics, tumour microenvironment

## Abstract

**Background:**

Cardiac myxoma (CM) is the most common (58%–80%) type of primary cardiac tumours. Currently, there is a need to develop medical therapies, especially for patients not physically suitable for surgeries. However, the mechanisms that shape the tumour microenvironment (TME) in CM remain largely unknown, which impedes the development of targeted therapies. Here, we aimed to dissect the TME in CM at single‐cell and spatial resolution.

**Methods:**

We performed single‐cell transcriptomic sequencing and Visium CytAssist spatial transcriptomic (ST) assays on tumour samples from patients with CM. A comprehensive analysis was performed, including unsupervised clustering, RNA velocity, clonal substructure inference of tumour cells and cell–cell communication.

**Results:**

Unsupervised clustering of 34 759 cells identified 12 clusters, which were assigned to endothelial cells (ECs), mesenchymal stroma cells (MSCs), and tumour‐infiltrating immune cells. Myxoma tumour cells were found to encompass two closely related phenotypic states, namely, EC‐like tumour cells (ETCs) and MSC‐like tumour cells (MTCs). According to RNA velocity, our findings suggest that ETCs may be directly differentiated from MTCs. The immune microenvironment of CM was found to contain multiple factors that promote immune suppression and evasion, underscoring the potential of using immunotherapies as a treatment option. Hyperactive signals sent primarily by tumour cells were identified, such as MDK, HGF, chemerin, and GDF15 signalling. Finally, the ST assay uncovered spatial features of the subclusters, proximal cell–cell communication, and clonal evolution of myxoma tumour cells.

**Conclusions:**

Our study presents the first comprehensive characterisation of the TME in CM at both single‐cell and spatial resolution. Our study provides novel insight into the differentiation of myxoma tumour cells and advance our understanding of the TME in CM. Given the rarity of cardiac tumours, our study provides invaluable datasets and promotes the development of medical therapies for CM.

## Graphical abstract

1. We present the first comprehensive characterisation of the TME in CM at single‐cell and spatial resolution.

2. Myxoma tumour cells included EC‐like and MSC‐like tumour cells, with the former potentially being differentiated directly from the latter.

3. The immune microenvironment of CM contained multiple factors that promote immune evasion, highlighting the potential of using immunotherapies as a treatment option.

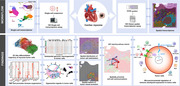

## 1 INTRODUCTION

Primary cardiac tumours (PCTs) are extremely rare, ranging from.001% to.03% in prevalence.^1^ Cardiac myxoma (CM) stands as the most prevalent type among PCTs in adults, accounting for 58% to 80% of cases.^1^ CM usually develops in the atria and has been histologically defined as a benign neoplasm. However, the designation of benignity may underestimate its potentially devastating impact on the patient. CM may cause life‐threatening consequences, such as stroke and heart failure.^2^ As of now, the sole definitive treatment for CM is surgical resection. Nonetheless, there is a need to develop targeted medical therapies to mitigate the tumour progression, especially in patients who are not physically suitable for surgeries.^3^

Histologically, tumour cells of CM typically form perivascular ring structures, which are embedded in a mucopolysaccharide myxoid matrix.^4^, ^5^ Histopathological analysis indicates that vascular and endothelial differentiation may be the typical paths of myxoma tumour cells,^6^ although other lineages, such as neural, muscular, and chondroid lineages, have also been reported.^3^ Therefore, it has been postulated that myxoma tumour cells may originate from multipotent mesenchymal cells.^7^ However, the histogenesis of CM has been uncertain until a recent study proposed that atrial myxoma is initiated from c‐kit^+^ CD31^−^ CD45^−^ cardiac progenitor/stem cells.^8^ Nevertheless, we still lack a systematic understanding of the differentiation trajectory and transcriptomic heterogeneity of myxoma tumour cells.

The intricate ecosystem of the tumour microenvironment (TME) encompasses tumour cells, infiltrated immune cells, other tissue‐resident cell types, and noncellular components, which interact with each other and collectively influence tumour progression.^9^ However, factors shaping the TME in CM remain poorly understood, which hinders the understanding of the mechanisms underlying tumour progression and impedes the development of targeted therapies. Unlike conventional bulk‐based expression profiling technologies, single‐cell RNA sequencing (scRNA‐seq) enables precise dissection of the TME.^10^ In addition, spatial transcriptomic (ST) profiling is a powerful technique that provides physical location information not captured in scRNA‐seq data, thus greatly expanding our ability to understand the TME underlying tumour progression.^11^

In this study, we sought to dissect the TME in CM at single‐cell and spatial resolution. Our analyses provide novel insight into the differentiation of myxoma tumour cells and advance our understanding of the TME in CM.

## 2 MATERIALS AND METHODS

### 2.1 Study subjects and tumour tissue collection

For scRNA‐seq, we enrolled CM patients (*n*  =  11) who had undergone surgical resection at Fuwai Hospital, the Chinese Academy of Medical Sciences (Table S1). The diagnosis of CM for each patient was confirmed histopathologically. In addition, there were no signs to support a diagnosis of the Carney complex. CM tissues were isolated during surgical resection. Tissues of nine patients were immediately placed in precooled saline for tissue dissociation. Additionally, formalin‐fixed paraffin‐embedded (FFPE) tissue blocks from three of the nine enrolled patients were prepared and subjected to ST assays. Therefore, samples from three patients had both scRNA‐seq and ST data. Tissue blocks from two additional patients were also subjected to ST assays.

### 2.2 Single‐cell suspension preparation

Fresh CM tissue was dissected into small fragments, and subjected to a single‐cold phosphate‐buffered saline (PBS) wash. The tissue fragments were then transferred to a 15 mL centrifuge tube with a 10 mL enzyme mixture (130‐095‐929, Miltenyi Biotec) prepared according to the manufacturer's instructions (9.35 mL DMEM, 50 μL Enzyme A, 200 μL Enzyme R, and 400 μL Enzyme H). Then, the tube was placed on a table concentrator for 30 min (37°C, 100×*g*). Single‐cell suspensions obtained underwent filtration through strainers of 100 and 40 μm to eliminate cellular debris and tissue. Afterward, the cell suspensions underwent centrifugation, with subsequent double washes at 500×*g* for 3 min at 4°C. The resulting pellets were then resuspended in PBS. Measurement of cell concentration and viability was conducted using a Countstar Rigel S3 cell counter (Alit Biotech, Shanghai).

### 2.3 Single‐cell transcriptomic sequencing

The single‐cell suspension was applied to a Chromium Controller (10X Genomics). Employing the Chromium Next GEM Single Cell 3ʹ Reagent Kits v3.1, libraries for single‐cell gene expression were prepared. Subsequently, sequencing of the libraries occurred on a system of Illumina NovaSeq 6000.

### 2.4 Preprocessing of the scRNA‐seq data

The sequencing reads were processed using the CellRanger software suite (v6.1.1), which aligned the reads to the human reference genome (version: refdata‐gex‐GRCh38‐2020‐A) and finally produced a gene–barcode matrix. For quality control, the matrix was then imported into the R package Seurat (v4.3.0).^12^ To eliminate genes identified as a result of random noise, those with counts in fewer than three cells were excluded. To eliminate cells of suboptimal quality, filtration was performed using criteria such as the number of genes, the count of unique molecular identifiers (UMIs), the proportion of ribosomal genes, and proportion of mitochondrial genes. The thresholds applied to each sample are provided in Table S2. To further remove doublets, cells were excluded if the predicted doublet scores by Scrublet (v0.2.3)^13^ > .3.

### 2.5 Normalisation, integration, dimensional reduction and clustering of the scRNA‐seq data

The UMI count for each cell was normalised to 10 000 and subsequently subjected to log transformation. With the “vst” method, highly variable genes were selected for each sample. To address potential batch effects, an integration of cells from all samples was performed using canonical correlation analysis. Additionally, unwanted sources of variation, including UMI count, gene number, cell cycle score, and the proportion of mitochondrial genes, were mitigated through regression using linear models. Subsequently, the scaled data underwent principal component analysis (PCA). The initial 30 PCA components were employed to generate a shared nearest neighbour (SNN) graph. The resulting SNN graph was then embedded in a two‐dimensional space with Uniform Manifold Approximation and Projection (UMAP). The original Louvain algorithm was used to perform the clustering of cells. All analyses mentioned above were carried out using Seurat (v4.3.0).^12^

### 2.6 Differential gene expression analysis

Differentially expressed genes between endothelial cell (EC)‐like tumour cells and left atrial ECs of normal hearts, as well as between mesenchymal stroma cell (MSC)‐like tumour cells of CM and left atrial fibroblasts of normal hearts were detected using the R package DEsingle (v1.18.0).^14^ The significance criteria were as follows: adjusted *p*‐value < .05, absolute log2 fold change > 1, and categorisation as “general differential expression” (the gene is different between conditions in terms of both the fraction of real zeros and expression abundance). The single‐cell/nucleus expression data of normal hearts from 14 individuals^15^ were downloaded from the Heart Cell Atlas database (https://www.heartcellatlas.org/v1.html). The file names of the downloaded data were “hca_heart_vascular_raw.h5ad” and “hca_heart_fibroblasts_raw.h5ad”. To detect the gene signature of subclusters, the function “FindAllMarkers” of Seurat was used (test.use = “bimod”, min.pct = .1, logfc.threshold = .25 and return.thresh = .01).

### 2.7 Functional enrichment analysis

Functional enrichment of a set of genes was conducted using CluoGO (v2.5.9)^16^ under default settings. A Bonferroni‐corrected *p*‐value threshold of < .05 was employed to determine statistical significance.

### 2.8 Copy number profile inference and clonal substructure analysis

The R package SCEVAN (v1.0.1)^17^ was used to infer the copy number profile based on scRNA‐seq or ST data under default settings. Cellular classification (tumour or normal cells) and intratumour clonal substructure inference within a single sample were conducted using the pipeline “pipelineCNA”. Intertumoural comparison among multiple samples was performed using the pipeline “multiSampleComparisonClonalCN.”

### 2.9 High‐dimensional weighted gene co‐expression network analysis

The R package hdWGCNA (v0.2.04),^18^ which is compatible with the high‐dimensional scRNA‐seq data, was utilised to conduct weighted gene co‐expression network analysis with default settings.

### 2.10 RNA velocity analysis

RNA velocity analysis was carried out using scVelo (v0.2.4).^19^ In brief, the quantification of spliced and unspliced mRNA expressions in each cell for every sample was performed using the tool Velocyto (v0.17.17).^20^ Then, the first‐ and second‐order moments were calculated with the first 30 PCA components. RNA velocities were estimated using a dynamical model and velocity graphs were constructed. In the end, the velocities were visualised in the form of streamlines. Genes with high likelihoods in the dynamic model were considered potential key genes.

### 2.11 10x Genomics visium CytAssist spatial transcriptomic assay

The isolation of total RNA from FFPE tissue blocks was achieved using the RNeasy FFPE kit (73504, Qiagen). The quality assessment of the extracted RNA was performed by calculating DV200. Tissue sections passing the quality control (DV200 > 50%) were subjected to ST assay by following the protocol of Visium CytAssist Spatial Gene Expression for FFPE (10x Genomics). Briefly, the tissue sections were placed on Sigma–Aldrich Poly Prep Slides and dried overnight. Then, the slides underwent incubation at 60°C for a duration of 2 h followed by deparaffinisation. Subsequently, the sections were stained with H&E and imaged at 20× magnification in brightfield using a Leica Aperio Versa 8 whole‐slide scanner (Leica, Germany). For the H&E‐stained sections, decrosslinking was carried out immediately and then probe panels covering the entire human transcriptome were incorporated. After the probe pairs were hybridised with their target transcripts, the slides were placed on a Visium CytAssist instrument for permeabilisation and RNase treatment. Subsequently, the ligated probes underwent hybridisation with spatially barcoded oligonucleotides. In the final step, libraries for STs were prepared from the probes and subsequently sequenced on a system of Illumina NovaSeq 6000.

### 2.12 Spatial transcriptomic data processing

The tool kit Space Ranger (v2.0.0) was used to perform tissue/fiducial detection, read alignment, and UMI counting of the ST data of each section. The obtained gene–spot matrices were then imported into Seurat for downstream analysis. Briefly, the expression was normalised for each section using the SCTransform procedure. Then, the data from different sections were integrated using the canonical correlation analysis procedure to correct for technical differences. After PCA reduction, the first 20 PCA components were used to construct an SNN graph. The clustering of spots was carried out using the original Louvain algorithm. To integrate the ST data with the scRNA‐seq data, the anchor‐based integration workflow of Seurat was applied to assign each spot a prediction score for each subcluster that were obtained from the single‐cell analysis. The predicted composition of cell types and subclusters identified by the scRNA‐seq data was visualised at the spot level for each CM tissue section using the function “deconvolution_plot” in the Python package stLearn (v0.4.12).^21^ Spatially proximal cell–cell communition inference from the spatial data was performed using CellChat v2^22^ under default settings.

### 2.13 Intercellular communication analysis based on scRNA‐seq data

CellChat (v1.6.1)^23^ was employed to deduce ligand–receptor interactions between cell types. In brief, overexpressed receptors or ligands in each cell type were identified, and the potential strength of interactions between any two cell types was determined using a probability value of communication. To identify significant interactions, a permutation test was executed with a significance threshold set at *p*‐value < .05. This involved random permutations of cell‐type labels, followed by the recalculation of the communication probability.

### 2.14 Single‐molecule fluorescence **in situ** hybridisation

We performed single‐molecule fluorescence in situ hybridisation (smFISH) on FFPE CM sections of 5 μm thickness using the RNAscope Multiplex Fluorescent Reagent Kit v2 (323100, Advanced Cell Diagnostics). Fluorescence signals were scanned with the Vectra Polaris pathology imaging system (PerkinElmer, USA). The target gene probes were as follows: Hs‐CDH5‐C2 (437451‐C2, Advanced Cell Diagnostics), Hs‐NPR3‐C3 (431241‐C3, Advanced Cell Diagnostics), Hs‐CCDC3‐C1 (1220531‐C1, Advanced Cell Diagnostics), Hs‐MIA (533581, Advanced Cell Diagnostics), Hs‐PDGFRA‐C3 (604481‐C3, Advanced Cell Diagnostics), Hs‐CPE‐C2 (454101‐C2, Advanced Cell Diagnostics) and Hs‐TSPAN8‐C2 (816551‐C2, Advanced Cell Diagnostics).

### 2.15 Inferring microenvironmental regulation of immune checkpoint expression in tumour cells

scMLnet (v.0.1.0)^24^ was used to infer intercellular communications between CM tumour cells (receivers) and other cell types (senders), as well as intracellular regulatory networks of immune checkpoint expression in CM tumour cells. Default parameters were used.

### 2.16 Statistical analysis

All statistical analyses were performed using R. Differences between two groups were compared using a two‐tailed Wilcoxon rank sum test.

## 3 RESULTS

### 3.1 The single‐cell transcriptomic landscape of cardiac myxoma

**FIGURE 1:**
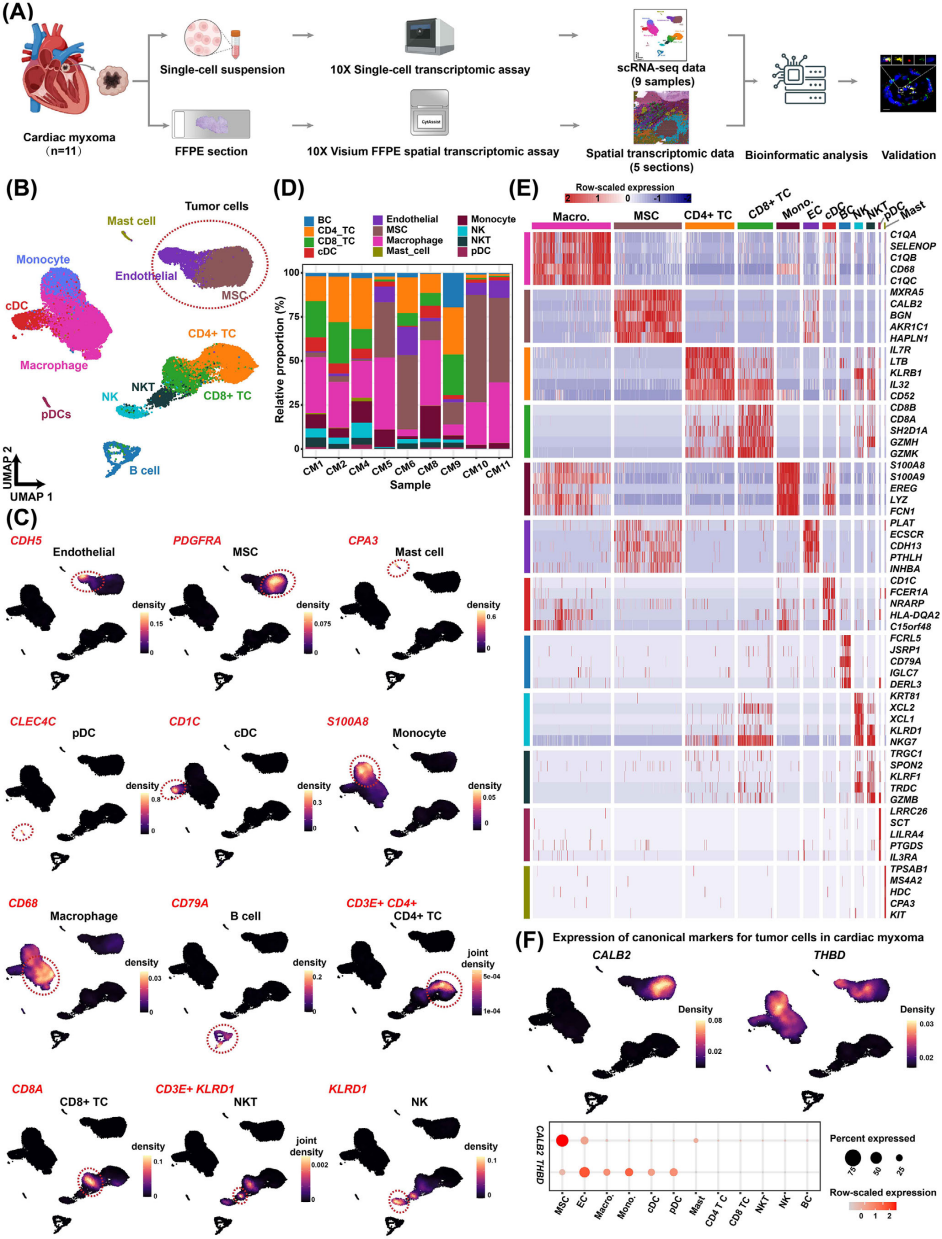
Single‐cell transcriptomic landscape of CM. (A) Schematic representation of the experimental procedure. CM tissues from patients (*n* = 11) were collected during surgical resection. Fresh samples from nine patients were individually subjected to scRNA‐seq, and FFPE tissue sections from five patients were subjected to ST assays. Three patients had both single‐cell and ST data available and two patients had only ST data. (B) UMAP visualisation of cellular identity. (C) Expression distribution of the marker gene(s) for each cell type. The visualisation was enhanced by gene‐weighted density estimation using the R package Nebulosa to recover the signal from dropped‐out features. (D) Relative proportion of each cell type in each sample. (E) Heatmap showing the expression of representative signature genes for each cell type. (F) UMAP (upper panel) and dot plots (lower panel) showing the expression of the canonical markers *CALB2* and *THBD* for myxoma tumour cells in each cellular cluster. BC, B cell; cDC, conventional plasmacytoid dendritic cell; EC, endothelial cell; MSC, mesenchymal stroma cell; Macro, macrophage; Mono, monocyte; NK, natural killer cell; NKT, natural killer T cell; pDC, plasmacytoid dendritic cell; TC, T cell; CM, cardiac myxoma; ST, spatial transcriptomic; scRNA, single‐cell RNA; UMAP, Uniform Manifold Approximation and Projection.

We performed scRNA‐seq of tumour tissue samples from nine patients with CM (Figure 1A and Table S1). After quality filtering, 34 759 cells were clustered into 12 clusters (Figure 1B and Table S2). According to the expression of canonical markers for each cell type, these clusters were assigned to different cell types (Figure 1C). Tumour‐infiltrating immune cells accounted for the majority (73.4%) of the captured cells. The nonimmune counterpart involved two cellular identities: ECs (marked by *CDH5*) and MSCs (marked by *PDGFRA*). Notably, ECs and MSCs were located closely adjacent to each other in the UMAP space (Figure 1B), instead of being in separate clusters as typically observed in normal tissues. This implies a potential transition between them. The cellular composition varied significantly among samples, reflecting high intertumoural heterogeneity (Figure 1D). The expression signature for each cell type in CM was identified (Figure 1E and Table S3).

Next, to identify myxoma tumour cells, we examined the expression of canonical diagnostic markers for myxoma tumour cells, including *CALB2* (encoding calretinin) and *THBD* (encoding thrombomodulin; Figure 1F).^3^ Notably, *CALB2* was highly expressed in the MSC cluster but was also expressed in the EC cluster. Moreover, we examined the expression of more previously reported markers^3^, ^6^ across all cell types (Figure S1). Many were expressed in both the EC and MSC clusters, such as *HAND2*, *GATA4*, *SOX9*, *FGF2* and *CD34*. Together, our data showed that the EC and MSC clusters may represent two phenotypic states of myxoma tumour cells, hereafter referred to as EC‐like tumour cells (ETCs) and MSC‐like tumour cells (MTCs).

### 3.2 **Tumour**/normal cell classification and clonal substructure inference based on copy number profiles

**FIGURE 2:**
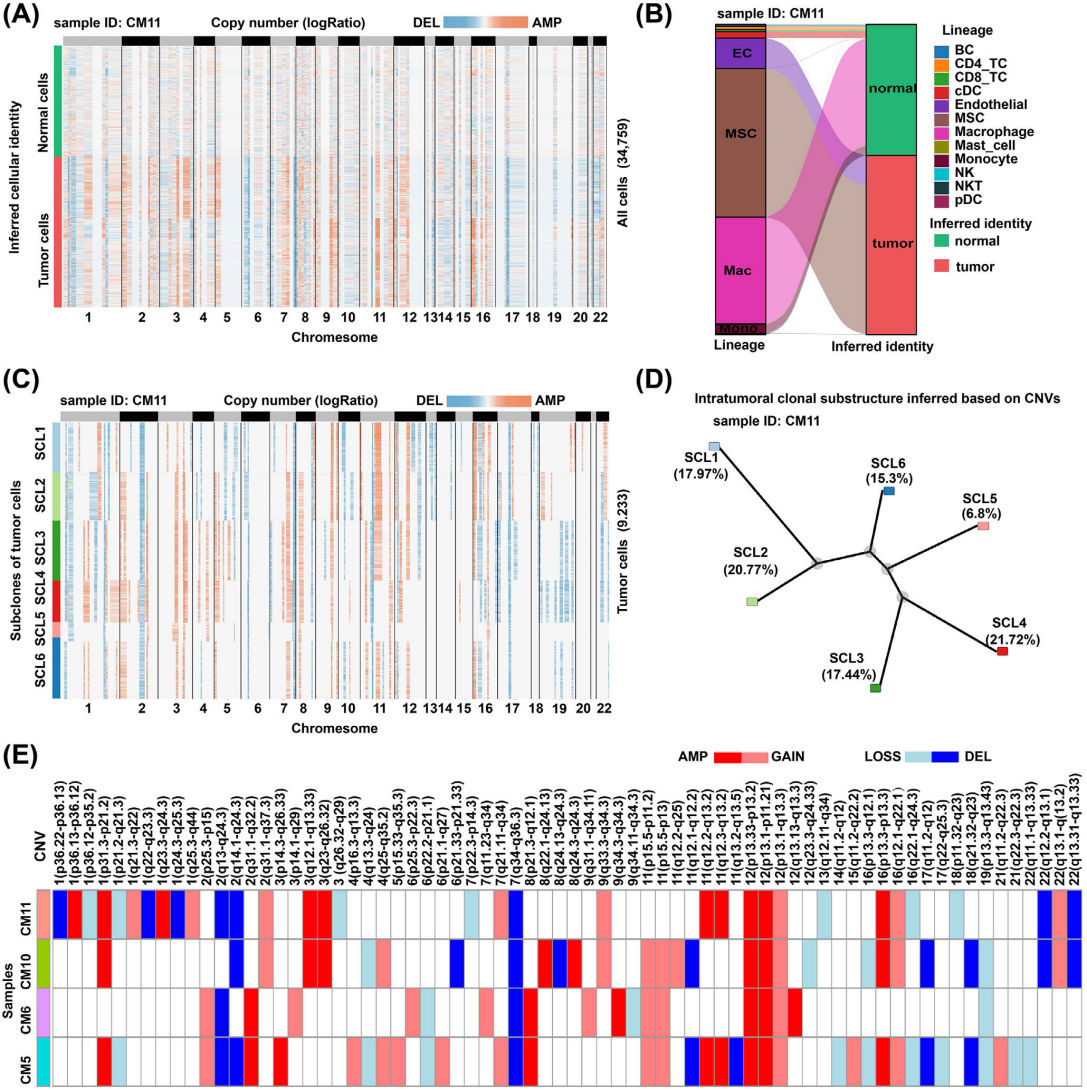
Tumour/normal cell classification and clonal substructure inference in CM based on copy number profiles inferred by the scRNA‐seq data. (A) Classification of myxoma cells as tumour cells and normal cells based on the copy number profile in sample CM11. (B) Alluvial plot showing that the myxoma cells with EC or MSC identities were classified as tumour cells in sample CM11. (C) Copy number profile of each subclone in tumour cells of sample CM11. (D) Phylogenetic tree showing the intratumoural clonal substructure of sample CM11. (E) OncoPrint‐like plot highlighting intertumoural heterogeneity in CM. The above analyses were performed using the method implemented in the R package SCEVAN. Only four samples were considered because they had a relatively high proportion of tumour cells. In A–D, the results are shown for a representative sample CM11. AMP, amplification; CNV, copy number variation; CM, cardiac myxoma; DEL: deletion; scRNA‐seq, single‐cell RNA‐seq.

To confirm the tumour identity of ETCs and MTCs, we applied the tumour /normal cell classification methods implemented in the R package SCEVAN, which infers copy number profiles based on single‐cell transcriptomic data. Notably, the analysis uncovered substantial copy number variations (CNVs), by which the cells were classified as either tumour cells or normal cells (Figure 2A). While immune cells were expectedly classified as normal cells, ETCs and MTCs were classified as tumour cells (Figure 2B), which reinforced our inference above based on marker gene expression: myxoma tumour cells harboured endothelial or mesenchymal identities. Furthermore, significant intratumour genomic heterogeneity was observed and a complex clonal substructure was uncovered (Figure 2C,D and Figures S2–S5). For example, six subclones of tumour cells were identified with different CNV profiles in sample CM11. In addition to some shared CNVs among samples, there were many sample‐specific CNVs in tumour cells, reflecting significant intertumoural heterogeneity (Figure 2E). Together, similar to malignant tumours, tumour cells in CM also demonstrated substantial genomic rearrangements and exhibited significant intra‐ and intertumoural genomic heterogeneity.

### 3.3 Transcriptomic heterogeneity of the **mesenchymal stroma cell**‐like tumour cells in cardiac myxoma

**FIGURE 3:**
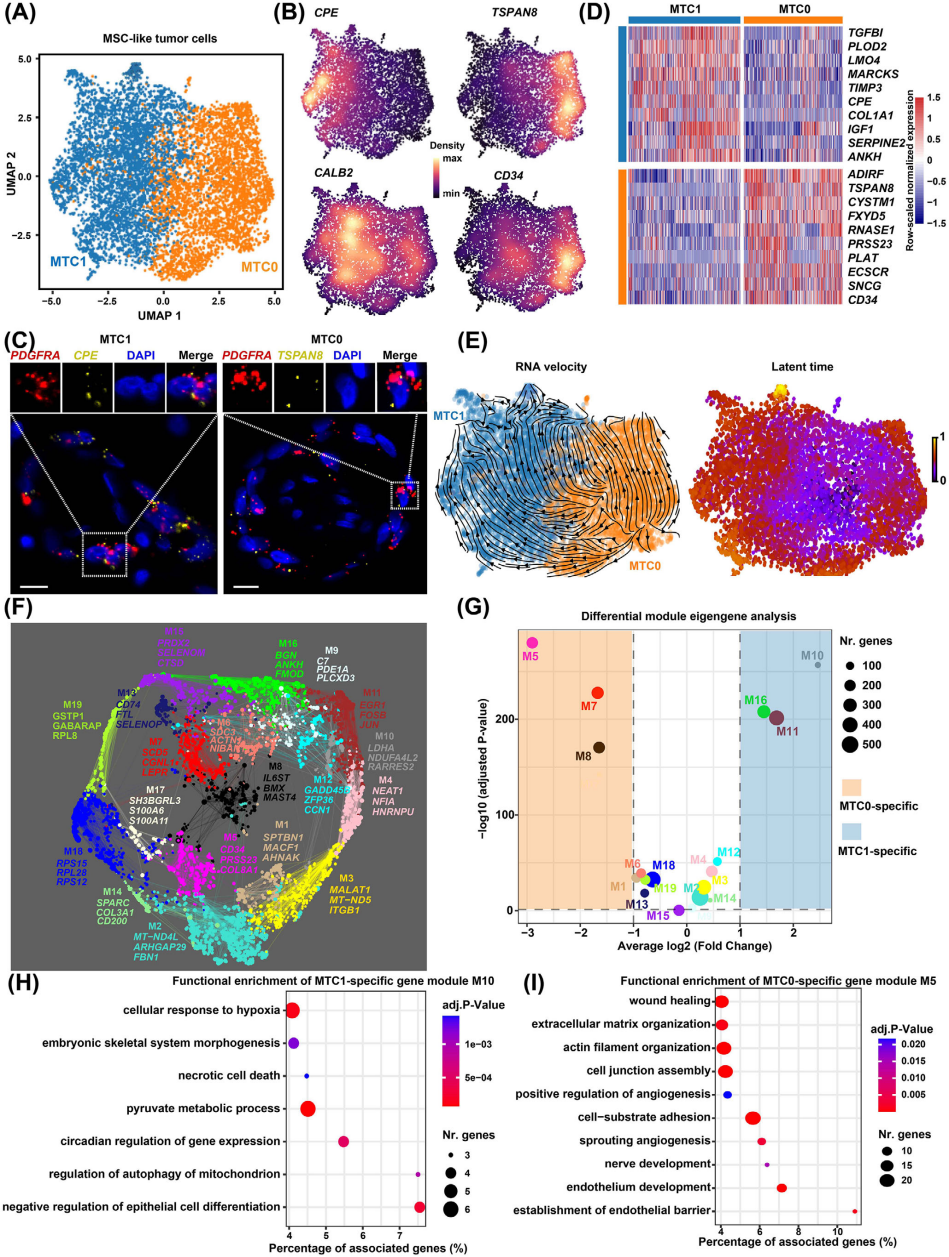
Transcriptomic heterogeneity of the MSC‐like tumour cells in CM. (A) The subclusters of MSC‐like tumour cells identified by secondary clustering. (B) Expression distribution of representative marker genes. (C) The presence of the two major subclusters confirmed by smFISH. Scale bar: 10 μm. Ring‐like structures formed by tumour cells are displayed. (D) Heatmap showing the expression of the top signature genes for each subcluster. (E) Differentiation trajectory (left panel) and latent time (right panel) in MSC‐like tumour cells inferred by RNA velocity analysis. The arrows indicate differentiation directions. (F) Gene co‐expression network of MSC‐like tumour cells. Each dot denotes a single gene. The dot is colour‐coded by the gene module. The dot size is scaled by the gene's eigengene‐based connectivity (kME). The top three genes ranked by kME of each module are shown. (G) Subcluster‐specific gene modules identified by differential module eigengene analysis. Two‐tailed Wilcoxon rank sum test. The significance threshold was set to an absolute log2 (fold change) value > 1 and a *p*‐value adjusted for multiple testing < .05. (H) Functional enrichment of subcluster MTC1‐specific gene module M10. (I) Functional enrichment of subcluster MTC0‐specific gene module M5. In (H,I), only representative gene ontology terms for biological processes are shown. Hypergeometric tests were performed for the functional enrichment analysis using ClueGO (significance threshold: *p*‐value adjusted for multiple testing < .05). CM, cardiac myxoma; MSC, mesenchymal stroma cell; smFISH, single‐molecule fluorescence in situ hybridisation.

To decipher the transcriptomic heterogeneity of the MSC‐like tumour cells, we performed secondary clustering and identified two subclusters (Figure 3A and Table S4). Subcluster MTC1 was marked by the expression of *CPE*, which encodes carboxypeptidase E (Figure 3B). Subcluster MTC0 was characterised by high expression of *TSPAN8* (encoding tetraspanin 8), which is overexpressed in many cancers,^25^ and *CD34*, a common marker for diverse progenitors.^26^ The presence of the two subclusters MTC0 and MTC1 was confirmed by smFISH (Figure 3C). Functional enrichment analysis of the signature genes for each subcluster (Figure 3D) was performed. Notably, the signature genes of MTC1 were significantly enriched for connective tissue development‐related gene ontology (GO) terms (e.g., “mesenchyme development” and “skeletal system development”) and terms associated with degenerative changes such as necrosis (e.g., “positive regulation of fibroblast apoptotic process”), calcification (e.g., “regulation of ossification”), and myxoid matrix production (e.g., “extracellular matrix organisation” and “glycosaminoglycan metabolic process”; Figure S6A and Table S5). These findings suggest that MTC1 may represent a differentiated state of tumour cells characterised by myxoid matrix production and degenerative changes. The signature genes of MTC0 were enriched for vasculature‐related terms such as “vasculature development” and “regulation of angiogenesis”, suggesting that MTC0 had properties of vasculature progenitors.

Moreover, RNA velocity analysis suggested a potential transition from MTC0 to MTC1 (Figure 3E), implying that MTC1 may represent a more differentiated/activated state relative to MTC0. To further characterise the functional properties of the subclusters and to identify functional gene modules associated with each subcluster, we performed co‐expression network analysis using hdWGCNA.^18^ A total of 19 gene modules were identified in the co‐expression network of MSC‐like tumour cells (Figure 3F and Figure S6D). Modules M10, M11 and M16 were significantly associated with subcluster MTC1, while modules M5, M7 and M8 were associated with MTC0 (Figure 3G). Notably, module M10 (*LDHA* as the top hub gene) was enriched for degenerative processes (e.g., hypoxia, necrosis and calcification)‐related terms (Figure 3H), while module M5 (*CD34* as the top hub gene) was particularly enriched for endothelium‐related terms (Figure 3I and Table S6).

### 3.4 Transcriptomic heterogeneity and differentiation dynamics of the endothelial cell‐like tumour cells in cardiac myxoma

**FIGURE 4:**
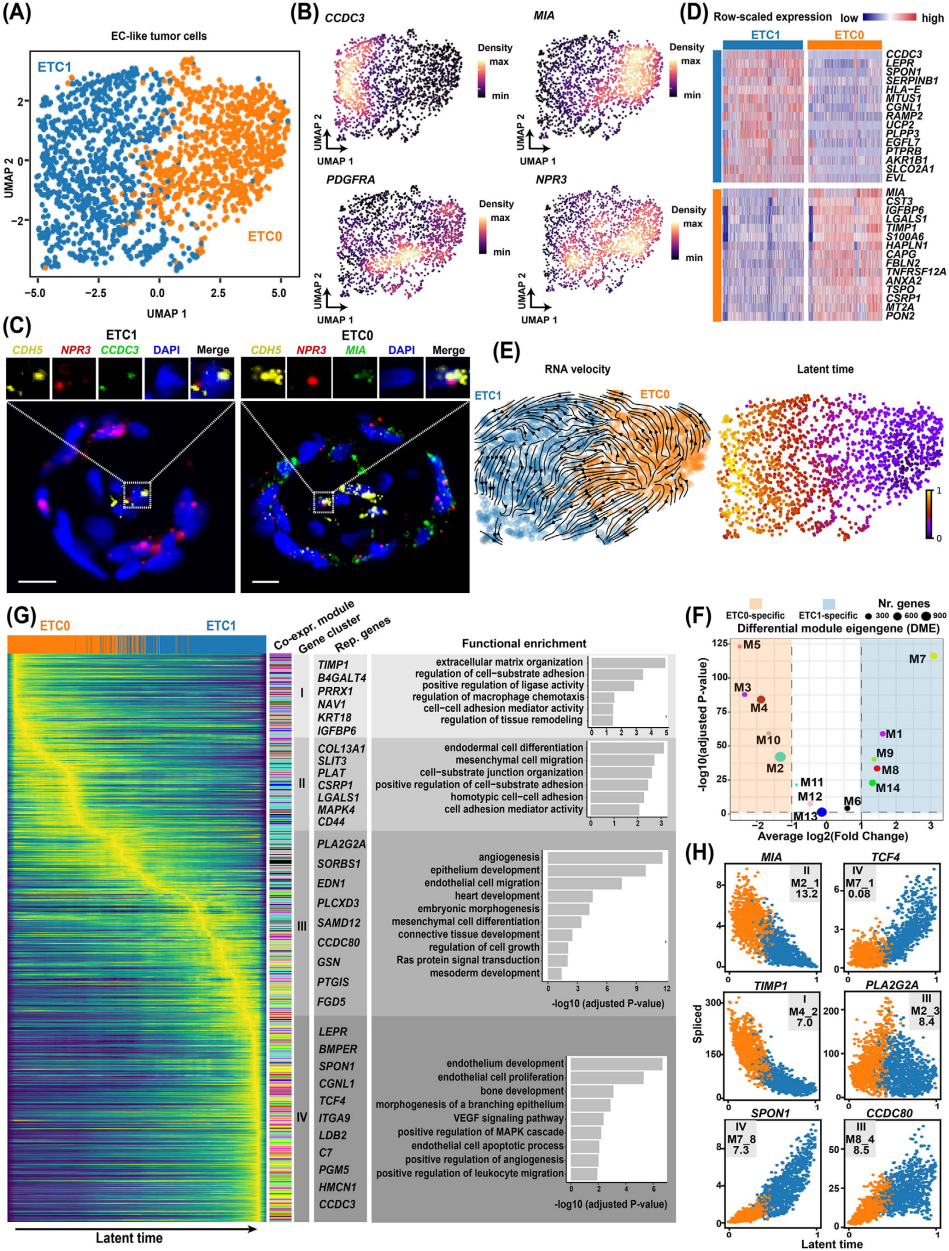
Transcriptomic heterogeneity and differentiation dynamics of the EC‐like tumour cells in CM. (A) Two subclusters of EC‐like tumour cells identified by secondary clustering. (B) Expression distribution of representative marker genes. (C) The presence of the two subclusters confirmed by smFISH. Scale bar: 10 μm. Ring‐like structures formed by tumour cells are displayed. (D) Heatmap showing the expression of the top signature genes for each subcluster. (E) Differentiation trajectory (left panel) and latent time (right panel) in EC‐like tumour cells inferred by RNA velocity analysis. (F) Subcluster‐specific gene modules identified by differential module eigengene analysis. Two‐tailed Wilcoxon rank sum test. The significance threshold was set to an absolute log2 (fold change) value > 1 and a *p*‐value adjusted for multiple testing < .05. (G) Heatmap showing expression dynamics during the differentiation of EC‐like tumour cells (ETC0‐to‐ETC1). Genes whose expression was dynamically changed over latent time were clustered into four gene clusters (I–IV). Representative genes and enriched gene ontology terms of each gene cluster are shown. The color of each module is consistent with that presented in (f). (H) Potential key genes driving the differentiation of EC‐like tumour cells. The annotation shown on the panel, for example, “II, M2_1, and 13.2″ on the panel of the gene *MIA*, denotes that *MIA* belongs to gene cluster II, and is also the top hub gene of gene module M2 with a log2 (fold change) of 13.2 in expression between EC‐like tumour cells in CM and left atrial ECs of healthy hearts. EC, endothelial cell; CM, cardiac myxoma; smFISH, single‐molecule fluorescence in situ hybridisation.

Compared with left atrial ECs in healthy hearts,^15^ EC‐like tumour cells in CM exhibited significant activation of various development‐related pathways (Figure S7 and Table S7), reflecting their large differences from normal ECs. Secondary clustering identified two subclusters, ETC1 (marked by *CCDC3*) and ETC0 (marked by *MIA*), in EC‐like tumour cells (Figure 4A,B and Table S8). Notably, few cells of the two subclusters expressed markers for human heart capillary ECs (e.g., *RGCC* and *CA4*), arterial ECs (e.g., *SEMA3G*), or venous ECs, for example, *ACKR1*,^15^ whereas both subclusters expressed the pan‐EC marker *CDH5* and markers for endocardial ECs (e.g., *NPR3* and *SMOC1*; Figure S8A), suggesting endocardial EC‐like differentiation of tumour cells. The presence of the two subclusters was confirmed by smFISH (Figure 4C). The signature genes of ETC1 (Figure 4D) were mainly enriched for terms related to differentiation, migration, and development of ECs (Figure S8B and Table S9), suggesting that ETC1 represented a more differentiated state of ETCs. Subcluster ETC0 was adjacent to MTCs in the UMAP space (Figure S9) and expressed the MSC marker *PDGFRA* (Figure S4B), reflecting that it might have progenitor properties. In support of this, RNA velocity analysis suggested a potential differentiation trajectory of ETC0‐to‐ETC1 (Figure 4E). Moreover, RNA velocity analysis suggested that ETCs may be directly differentiated from MTCs (Figure S10), supporting the view that ECs may be the typical destination of myxoma cell differentiation.^6^

In addition, co‐expression network analysis identified 14 co‐expression modules in the EC‐like tumour cells (Figure S8D,E and Table S10), many of which exhibited differences in expression activity between subclusters (Figure 4F). Transcriptomic dynamics during the differentiation were uncovered (Figure 4G and Table S11). To prioritise the potential key genes driving the differentiation, we set stringent criteria: the gene must be within the top 5% of genes ranked by likelihood in the RNA velocity dynamic model and be one of the hub genes of co‐expression modules (defined as the top 10 genes ranked by eigengene‐based connectivity). A total of six potential key genes were identified, including *MIA*, *TIMP1*, *SPON1*, *PLA2G2A*, *TCF4* and *CCDC80* (Figure 4H). Notably, *MIA*, *PLA2G2A* and *TIMP1* have previously been recognised as diagnostic markers of endothelial cell.^27^ The expression levels of *TCF4*, *SPON1* and *CCDC80* increased over the latent time, which may function in the activation/maintenance of endothelial cell‐related transcription programs.

### 3.5 T**umour** immune microenvironment in cardiac myxoma dissected at single‐cell resolution

**FIGURE 5:**
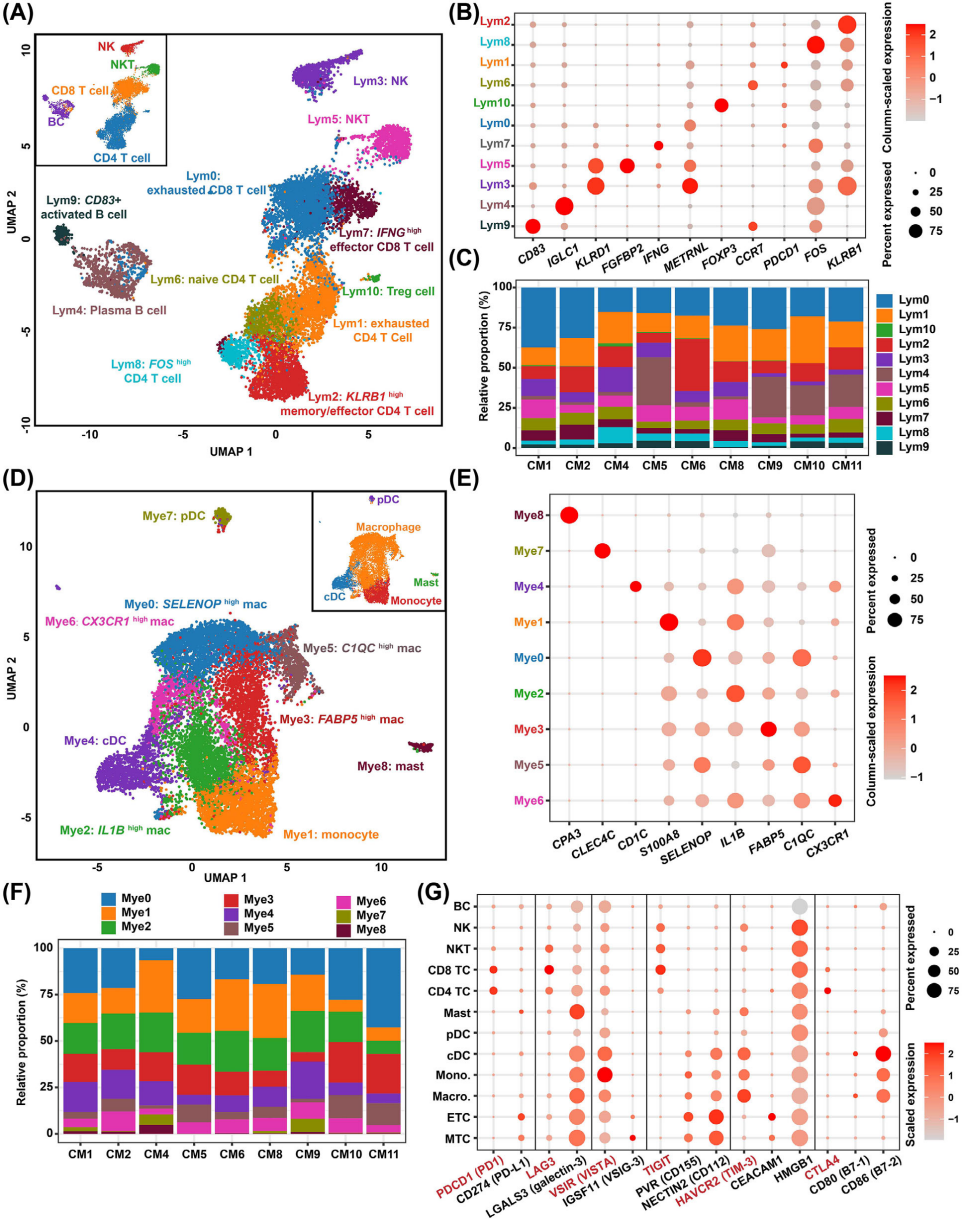
Tumour immune microenvironment in CM dissected at a single‐cell resolution. (A) The UMAP plot showing subclusters of lymphoid cells. The small panel shows cells color‐coded by the cell type. (B) The dot plot showing the expression of marker genes for each subcluster of lymphoid cells. (C) Relative proportion of each subcluster of lymphoid cells in each sample. (D) The UMAP plot showing subclusters of myeloid cells. The small panel shows cells color‐coded by cell type. (E) The dot plot showing the expression of marker genes for each subcluster of myeloid cells. (F) Relative proportion of each subcluster of myeloid cells in each sample. (G) The dot plot showing the expression of tumour ‐associated immune checkpoint receptors (in red) and ligands (in black). CM, cardiac myxoma; ETC, EC‐like tumour cell; MTC, MSC‐like tumour cell; UMAP, Uniform Manifold Approximation and Projection.

To dissect the immune microenvironment in CM, secondary clustering of tumour ‐infiltrating lymphoid or myeloid cells was performed. Exhausted T cell subclusters Lym0 and Lym1, characterised by the expression of inhibitory receptor genes such as *CTLA4*, *PDCD1* and *LAG3* ^28^ were identified in CD8+ and CD4+ T cells, respectively (Figure 5A, Figure S11A, and Table S12). In addition, naive CD4+ T cells (Lym6; marked by expression of *CCR7*, *SELL* and *LEF1*; Figure S11B) and regulatory T cells (Lym10; marked by *FOXP3*) were found (Figure 5B). Notably, the exhausted T cell subclusters (Lym0 and Lym1) accounted for a relatively high proportion of lymphoid cells (28%−49%) in each sample (Figure 5C), which were potential cellular targets of immunotherapies. Secondary clustering of myeloid cells uncovered five subclusters of macrophages (Figure 5D–F and Table S13). All of these subclusters represented M2‐like macrophages marked by the expression of *CD163* and *MRC1* (Figure S11C), which potentially contribute to tumour progression.^29^

Moreover, we examined the expression of genes encoding tumour‐associated immune checkpoint receptors and ligands across cell types (Figure 5G). Myxoma tumour cells, including ETCs and MTCs, expressed a relatively high level of genes encoding most immune checkpoint ligands, such as galectin‐3, PD‐L1, VSIG‐3, CD155, CD112 and CEACAM1. The corresponding receptors were primarily expressed either by T cells (e.g., PD1, LAG3, TIGIT and CTLA4) or macrophages/monocytes (e.g., VISTA and TIM‐3). Moreover, scMLnet was employed to deduce intracellular regulatory networks potentially activated through intercellular communications, governing the expression of inhibitory immune checkpoint ligands in CM tumour cells (Figure S12 and Table S14). Although scMLnet was unable to deduce intracellular regulatory networks for the expression of PD‐L1, VSIG‐3 and CD112, the regulatory networks of galectin‐3, CEACAM1 and CD155 were constructed. Notably, the results indicated that T cells, normally suppressed by binding with the inhibitory immune checkpoint ligands expressed by tumour cells, were also potentially involved in regulating the expression of the three immune checkpoint ligand genes in CM tumour cells. For example, extracellular signal ligands sent by T cells, such as OSM and MFNG, may bind with the receptors LIFR and NOTCH1, respectively, on the cell surface of MTCs. This binding may activate the intracellular expression of the transcription factor RUNX1, ultimately resulting in the upregulation of galectin‐3 (encoded by *LGALS3*).

Overall, the immune microenvironment of CM was found to contain multiple aspects that promote immune suppression and evasion.

### 3.6 Intercellular communication analysis uncovers hyperactive signals sent primarily by myxoma **tumour** cells

**FIGURE 6:**
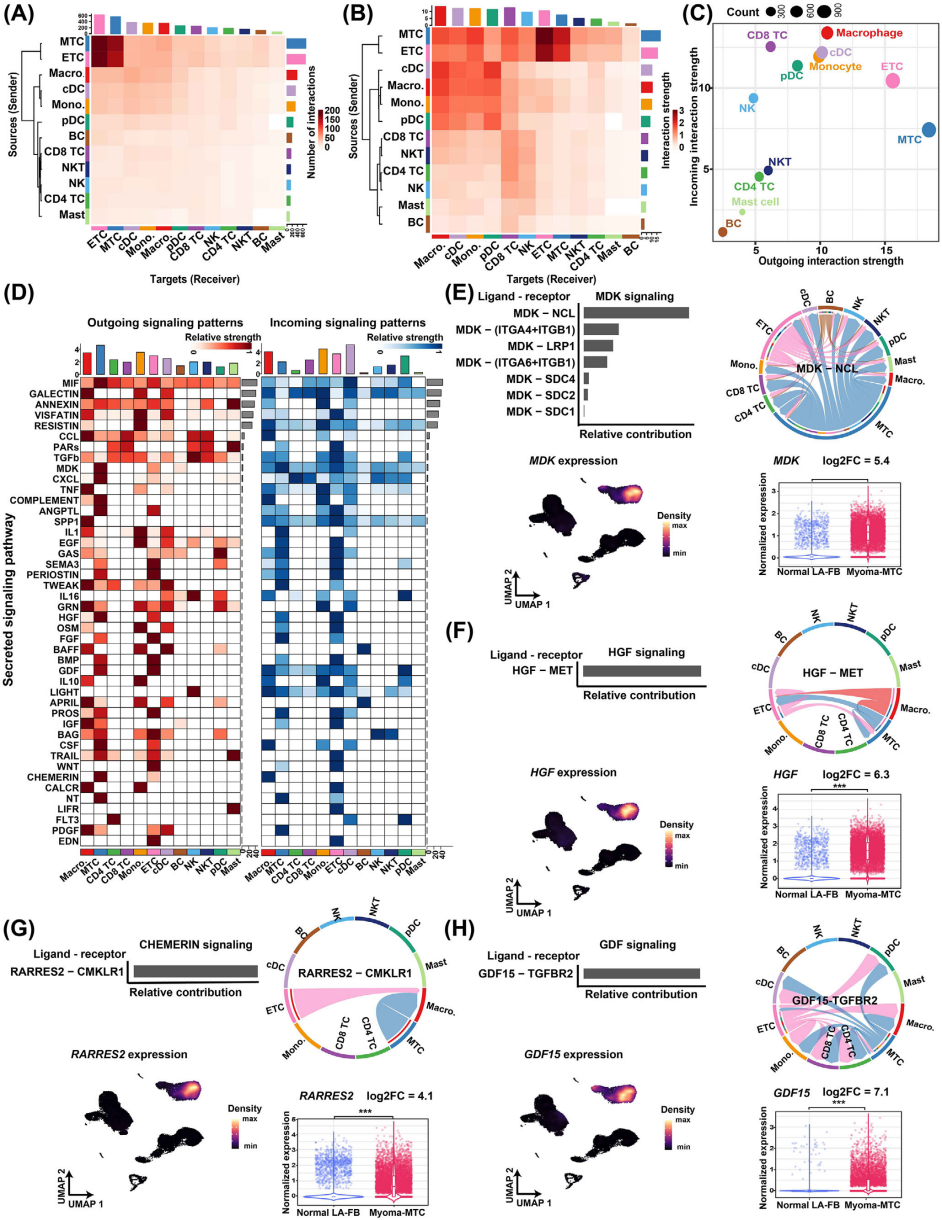
Intercellular communication analysis uncovers hyperactive signals sent primarily by myxoma tumour cells. (A) Heatmap showing the number of interactions among cell types inferred by CellChat. The bar denotes the total number of interactions that were sent (right) or received (top) by each cell type. (B) Heatmap showing interaction strength (communication probability) among cell types. The bar denotes the accumulated strength of interactions that were sent (right) or received (top) by each cell type. (C) The bubble plot showing the accumulated strength of interactions that were sent or received by each cell type. Dot size denotes the total number of interactions. (D) Strength of secreted signalling pathways sent (outgoing) or received (incoming) by each cell type. The top bars denote the accumulated strength of signals that were sent or received by each cell type. Right bars denote the accumulated strength of signals across all cell types. (E) MDK signal broadcast mainly by tumour cells. (F) HGF signal broadcast mainly by tumour cells. (G) CHEMERIN signal broadcast mainly by tumour cells. (H) GDF signal broadcast mainly by tumour cells. In (E–H), the bar plot shows the relative contribution of each ligand‐receptor pair in the pathway; the chord plot shows interaction strength among cell types for a specific ligand–receptor pair; the UMAP plot reflects the expression distribution of the ligand; and the violin plot shows the normalised expression of the ligand in MTCs of CM and left atrial fibroblasts of normal hearts (*p*‐value adjusted for multiple testing < .05, the differential expression analysis method implemented in the R package DEsingle). The expression data of normal hearts were downloaded from the Heart Cell Atlas database (https://www.heartcellatlas.org/v1.html). In the chord plot, the size of the inner bar is proportional to the signal strength received by the target cell types. ETC, EC‐like tumour cell; LA‐FB, left atrial fibroblasts; log2FC, log2 (fold change); MTC, MSC‐like tumour cell.

Next, we sought to infer the intercellular communication network in CM using CellChat. The tumour cell clusters (i.e., MTC and ETC) sent or received the largest number of signals (Figure 6A and Table S15), reflecting that the communication network was orchestrated primarily by the tumour cells. While the tumour cells exhibited the greatest strength (i.e., communication probability) of outgoing signals, macrophages had the greatest strength of incoming signals (Figure 6B,C). Furthermore, we identified the secreted signalling pathways that were activated in CM, as shown in Figure 6D. We focused on the hyperactive signals primarily sent by the tumour cells. For example, midkine growth factor (encoded by *MDK*) is known to be highly expressed in numerous malignant tumours and plays diverse roles in the tumour development.^30^ We found that *MDK* was primarily expressed by myxoma tumour cells, and was significantly overexpressed when compared to left atrial fibroblasts in normal hearts (Figure 6E and Table S16). The receivers of MDK signalling were found to involve nearly all cell types present in CM (Figure 6E), reflecting its multiple roles in driving tumour progression. Similarly, we observed other hyperactive signalling pathways in CM, such as HGF signalling, chemerin signalling, and GDF15 signalling (Figure 6F–H). These findings in CM were consistent with reports in many malignant tumours.^30^, ^31^, ^32^ This implies that therapeutic strategies targeting these signalling molecules in malignant tumours may have potential efficacy for the treatment of CM.

### 3.7 Spatial features of the subclusters, proximal cell–cell communication, and clonal evolution of myxoma **tumour** cells based on spatially resolved transcriptomics

**FIGURE 7:**
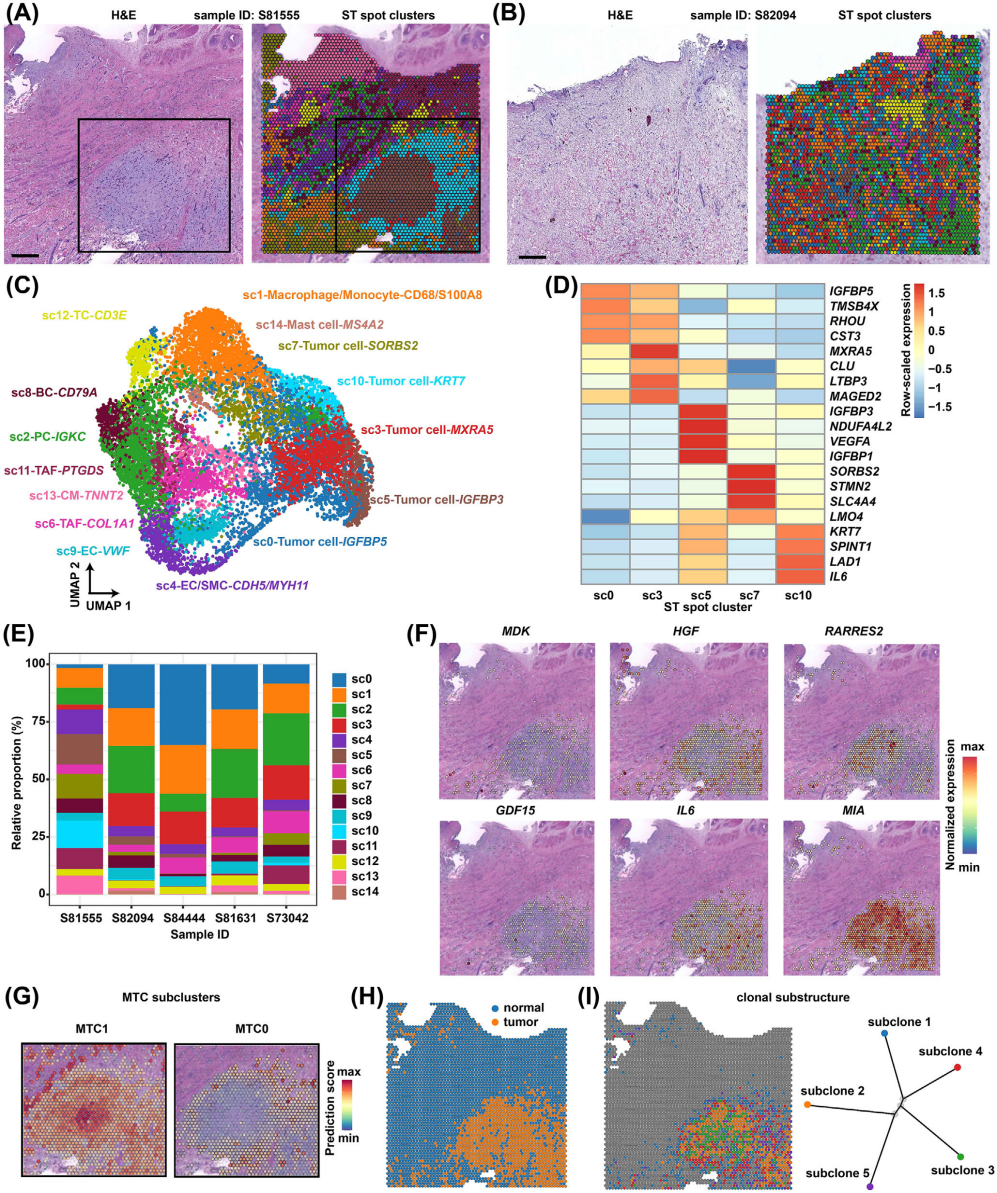
Spatial features of subclusters and clonal evolution of myxoma tumour cells based on spatially resolved transcriptomics. (A) H&E staining (left) and spatial transcriptome (ST) spot cluster distribution (right) for a section of sample S81555. Spots are colour‐coded by spot cluster. Scale bar: 1 mm. The black box indicates a region concentrated with ring‐ or cord‐like tumour cell structures. (B) H&E staining (left) and ST spot cluster distribution (right) for a section of sample S82094. Spots are colour‐coded by spot cluster. Scale bar: 1 mm. (C) The UMAP plot showing the clusters of ST spots of five sections from five patients. Each ST spot cluster was annotated with its major composition cell type. The colour of each cluster is consistent with that used in (A,B). (D) Heatmap showing the expression of the top features for each spot cluster annotated as tumour cells. (E) The relative proportion of each spot cluster in each sample. (F) Spatial distribution of the expression of representative genes encoding proteins secreted by tumour cells. Only the results on the section from sample S81555 are shown. (G) Predicted spatial distribution of MTC subclusters MTC1 and MTC0 on the section of sample S81555. The boxed region in (A) is displayed. The ST and scRNA‐seq data were integrated using the anchor‐based integration workflow of Seurat. (H) Copy number profile inferred by SCEVAN based on the ST data distinguishes tumour from normal cells on the section of sample S81555. (I) Spatial distribution (left) and clonal substructure (right) of subclones inferred by SCEVAN based on ST data on the section of sample S81555. BC, B cell; CM, cardiomyocytes; EC, endothelial cell; PC, plasma cell; SMC, smooth muscle cell; TAF, tumour‐associated fibroblast; TC, T cell; H&E, hematoxylin and eosin; UMAP, Uniform Manifold Approximation and Projection; MTC, MSC‐like tumour cell; scRNA‐seq, single‐cell RNA‐seq.

To characterise the TME in situ, we conducted ST assays on tissue sections from five patients (Tables S1 and S2). According to haematoxylin and eosin (H&E) staining, the tumour cells, which typically formed ring or cord‐like structures, were concentrated within a specific area of section S81555 (indicated by a black box; Figure 7A), while a diffuse distribution of the tumour cell structures was observed in most other sections (Figure 7B and Figure S13). The ST spots of all sections were clustered and the spot clusters were annotated to their major cell types based on the expression of lineage markers (Figure 7C, Figure S14, and Table S17). A total of five ST spot clusters were annotated to tumour cells (marked by relatively high expression of *CALB2*; Figure 7D). Notably, few macrophages/monocytes (sc1) and T cells (sc12) infiltrated the tumour cell‐enriched region, which was filled with abundant mucopolysaccharides (grey in H&E staining; Figure 7A), reflecting the secretion of an abundant myxoid matrix as a mechanism of immune escape for myxoma tumour cells. The relative proportions of the spot clusters varied significantly among different samples (Figure 7E). The expression of secreted protein‐coding genes, including *MDK*, *HGF*, *RARRES2*, *GDF15*, *IL6* and *MIA*, was concentrated in the tumour cell‐enriched region (Figure 7F), thus confirming the expression specificity of these signalling molecules.

To integrate the scRNA‐seq data, we adopted the anchor‐based integration workflow of Seurat, which enables probabilistic classification of cell types or subclusters for each ST spot. The predicted composition of cell types or subclusters identified by the scRNA‐seq data was visualised at the spot level for each CM tissue section (Figures S15–S19). In line with the composition analysis derived from scRNA‐seq data, MTC, particularly subcluster MTC1, was found to constitute the largest proportion within the tissue section. However, the predicted proportion based on the ST data generally exceeded that of scRNA‐seq due to the low resolution of ST spots. For instance, MTC was found to be the predominant cell type in both tissue section S81555 (comprising 63% of ST spots; Figure 1D) and the sample (CM6) from the same patient in scRNA‐seq (accounting for 42.2%; Figure S15). Notably, MTC1 was located close to the centre of the tumour cell‐enriched region filled with a thick myxoid matrix (Figure 7G and Figure S15), which explained why its signature genes were enriched for degenerative processes (e.g., hypoxia, necrosis and calcification). In contrast, MTC0, a less activated state of tumour cells, was located relatively distant from the centre. Next, spatially proximal cell–cell communication was inferred from the ST data of the representative section S81555 (Figure S20A) using the R package CellChat v2, which incorporates the spatial location information of cells.^22^ Tumour cells (comprising ETCs and MTCs) and macrophages constituted a dense module of communication (Figure S20B,C). Consistent with the results of single‐cell data (Figure 6), MDK signalling, HGF signalling, chemerin signalling, and GDF15 signalling were also observed to be hyperactive signals primarily sent by the tumour cells based on the ST data (Figure S20D). The spatially proximal cell–cell communication analysis consolidated the signalling networks of these pathways inferred from the single‐cell data (Figure S20E–G and Figure 6). For example, the ligand of the chemerin signalling pathway, RARRES2, was primarily sent by MTCs and was mainly received by macrophages (Figure S20E and Figure 6G), reflecting that tumour cells may regulate the phenotype of macrophages through the secretion of RARRES2.

In addition, the copy number profile inferred based on the ST data generally distinguished tumoural from normal spots (Figure 7H). The subclones of tumour cells were distributed in circles around the centre of the tumour cell‐enriched region (Figure 7I), reflecting phenotypic changes in CM tumour cells potentially through subclone evolution.

## 4 DISCUSSION

Developing targeted medical therapies for CM is essential to avoid surgeries, especially for patients who are not physically suitable for surgeries due to reasons such as advanced age, tumour location and risks of complications. Understanding the cellular composition, differentiation trajectory, regulatory network and cell‐to‐cell interactions in the TME is of fundamental importance for elucidating the mechanisms underlying tumour progression and developing targeted drugs. Here, we characterised the TME in CM at a single‐cell and spatial resolution for the first time. Based on marker gene expression and copy number profiles, we found that myxoma tumour cells encompassed two closely related phenotypic states, that is, ETCs and MTCs. The transcriptomic heterogeneity of tumour cells was found to be driven primarily by differentiation/activation stages. Based on RNA velocity and expression analyses, we found that ETCs may be directly differentiated from MTCs. Furthermore, potential key genes driving ETC differentiation were identified, including *MIA*, *TIMP1*, *SPON1*, *PLA2G2A*, *TCF4* and *CCDC80*. The immune microenvironment of CM was found to contain multiple factors that promote immune suppression and evasion, underscoring the potential of using immunotherapies as a treatment option for this tumour. The intercellular communication network in CM was found to be orchestrated primarily by tumour cells, and hyperactive signals sent primarily by tumour cells were identified, such as MDK, HGF, chemerin and GDF15 signalling. Finally, the spatial features of the subpopulation distribution and clonal evolution of myxoma tumour cells were determined based on spatially resolved transcriptomics.

Our current understanding of the cellular states of myxoma tumour cells has been derived primarily from studies that employed immunohistochemistry. Previously, it was noted that myxoma tumour cells were immunoreactive for endothelial markers such as CD31 and CD34.^3^, ^6^ These cells are spatially closely associated with vascular ECs and typically form myxomatous perivascular ring structures (single or multilayered) or pseudo‐vascular structures.^33^ These observations have led to the impression that vascular channels seem to arise from the myxomatous structures.^6^, ^34^ However, due to technical limitations, there is no evidence supporting the view that the ECs of the vascular channel in CM could be directly derived from myxoma tumour cells. Our single‐cell data supported the existence of two phenotypic states in myxoma tumour cells, namely, ETCs and MTCs, and that ETCs could be directly differentiated from MTCs. This finding was obtained based on multiple pieces of evidence, including the adjacency of ETCs and MTCs in the UMAP embedding (Figure 1B), RNA velocity (Figure 4E and Figure S10), and copy number profiles (Figure 2B). Moreover, ETCs were found to express markers for endocardial ECs (Figure S8A), indicating that endocardial EC‐like differentiation was the primary differentiation path for myxoma tumour cells. Therefore, drugs that target key genes involved in ETC differentiation are anticipated to have an impact on tumour growth and progression.

Malignant tumour cells are characterised by a high level of genomic instability, which results in frequent genomic rearrangements that contribute to cancer genome evolution.^35^ Genomic rearrangements have previously been reported in cases of sporadic CM based on cytogenetic analysis.^36^ However, the extent of intratumour genomic heterogeneity and the clonal substructure of myxoma tumour cells have been largely unknown. Here, we found that similar to malignant tumours, tumour cells in CM also demonstrated substantial genomic rearrangements, and exhibited significant intra‐ and intertumoural genomic heterogeneity (Figure 2). Moreover, the special feature of the clonal substructure of myxoma tumour cells was revealed for the first time: the subclones spread around the centre of the tumour cell‐enriched region (Figure 7I). Our results support the view that genomic overlap exists between benign and malignant tumours and that these two categories of tumours are rather similar at the mechanistic level.^37^ Therefore, the activation of cardiac stem/progenitor cells that initiates CM may be driven by somatic mutations.

The TME in CM was found to be similar to that in malignant tumours in several aspects. The intercellular communication network was orchestrated primarily by tumour cells (Figure 6). The hyperactive signals in many malignant tumours,^30^, ^31^, ^32^ such as MDK, HGF, chemerin and GDF15 signalling, were found to be sent primarily by myxoma tumour cells (Figure 6). The immune microenvironment of CM was found to contain multiple aspects that promote immune suppression and evasion, including the presence of abundant M2‐like macrophages and exhausted T cells, as well as high expression of most immune checkpoint ligands and receptors (Figure 5). These results underscore the potential of using targeted drugs and/or immunotherapies that are currently used for cancers as treatment options for CM. However, preclinical and clinical trials are essential to evaluate the efficacy of immunotherapies for the CM treatment.

The histomorphology of CM exhibits notable heterogeneity, especially in the inflammatory cells, myxoid stroma, necrosis, cystic changes, haemorrhage, calcification and other aspects.^38^ Among these factors, we believe that haemorrhage significantly impacts the proportion of tumour cells in single‐cell transcriptome experiments. This may account for the observed wide variation in the proportion of tumour cells among samples (Figure 1D). The current study was constrained by a small‐sample size due to the rarity of the tumour. More samples from different sampling sites of the same tumour and more patients may be needed to unveil the complete landscape of cellular heterogeneity. In addition, the ST analysis was limited by the resolution of the ST assay technology. The diameter of the capture spots (55 μm) generally exceeded that of the perivascular ring structures in tissue sections of CM, thereby impeding accurate spatial analysis of cellular subpopulations although deconvolution or integration analysis methods could be applied. A high‐resolution ST assay is thus warranted, especially for tissue sections with a diffuse distribution of the tumour cell structures.

## 5 CONCLUSIONS

Our study presents the first comprehensive characterisation of the TME in CM at both single‐cell and spatial resolution. Our analyses provide novel insight into the differentiation of myxoma tumour cells and advance our understanding of the TME in CM. Given the rarity of cardiac tumours, our study provides invaluable datasets and promotes the development of medical therapies for CM.

## AUTHOR CONTRIBUTIONS

Xuanyu Liu designed the project, analysed the data, and wrote the manuscript. Huayan Shen organised the sample collection and performed the experiments. Jinxing Yu, Fengming Luo, Tianjiao Li, Qi Li and Xin Yuan contributed to subject enrollment and specimen collections. Yang Sun and Zhou Zhou supervised the project.

## CONFLICT OF INTEREST STATEMENT

The authors declare no conflicts of interest.

## ETHICAL APPROVAL

All study procedures complied with the ethical regulations approved by the Ethics Committee of Fuwai Hospital, the Chinese Academy of Sciences (No. 2022‐1656). Written informed consent was received from each patient. The study conforms to the principles outlined in the Helsinki Declaration of 1975.

## Supporting information

Supporting information

Supporting information

## Data Availability

Raw sequencing data have been deposited in Genome Sequence Archive for humans (https://ngdc.cncb.ac.cn/gsa‐human/) and are available through the accession number HRA004062.
