# Supplementary material for: Resolving the heterogeneous tumour microenvironment in cardiac myxoma through single‐cell and spatial transcriptomics
Source: Clin Transl Med. 2024 Feb 6;14(2):e1581. doi: 10.1002/ctm2.1581 (PMC10844892; doi:10.1002/ctm2.1581)
Supplement: Supplementary file 1 — Supporting information [file CTM2-14-e1581-s001.docx]

**Supplemental Figures S1-S20 for the manuscript**

**Resolving** **the Heterogeneous Tumor Microenvironment in Cardiac Myxoma through Single-Cell and Spatial Transcriptomics**

Xuanyu Liu^1,2^, Huayan Shen^1,2^, Jinxing Yu^1,2^, Fengming Luo^1,2^, Tianjiao Li^1,2^, Qi Li^1,4^, Xin Yuan^1,4^, Yang Sun^1,2,3✉^, Zhou Zhou^1,2✉^

^1^State Key Laboratory of Cardiovascular Disease, Fuwai Hospital, National Center for Cardiovascular Diseases, Chinese Academy of Medical Sciences and Peking Union Medical College, Beijing, 100037， China;

^2^Beijing Key Laboratory for Molecular Diagnostics of Cardiovascular Diseases, Center of Laboratory Medicine, Fuwai Hospital, Beijing, 100037, China;

^3^Department of Pathology, Fuwai Hospital, Beijing, China; d: Department of Cardiovascular Surgery, Fuwai Hospital, Beijing, 100037, China

^4^Department of Cardiovascular Surgery, Fuwai Hospital, Beijing 100037, China

Xuanyu Liu and Huayan Shen contributed equally to this manuscript.

Correspondence: Yang Sun and Zhou Zhou, Center of Laboratory Medicine, Fuwai Hospital, Chinese Academy of Medical Sciences, Beijing, China. Email: sunyangyjs@sina.com, zhouzhoufuwai@126.org

## Funding Information

This work was supported by grants from the Chinese Academy of Medical Sciences Innovation Fund for Medical Sciences (2021-I2M-C&T-B-039) and the National High Level Hospital Clinical Research Funding (2023-GSP-RC-21, 2022-GSP-GG-6, and 2022-PUMCH-C-025).


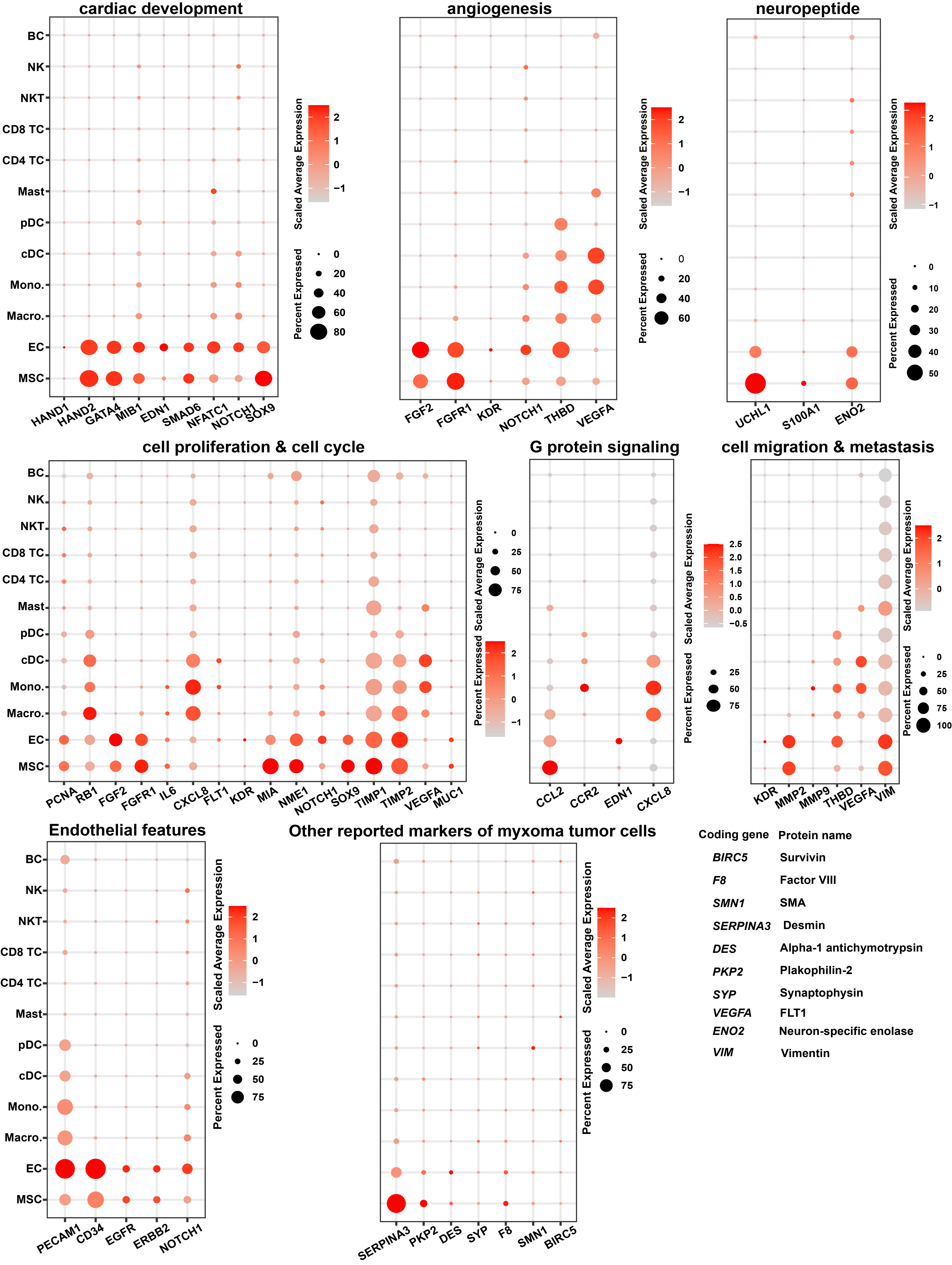


## Figure S1 Dot plots showing the expression of previously reported markers for cardiac myxoma tumor cells.

##
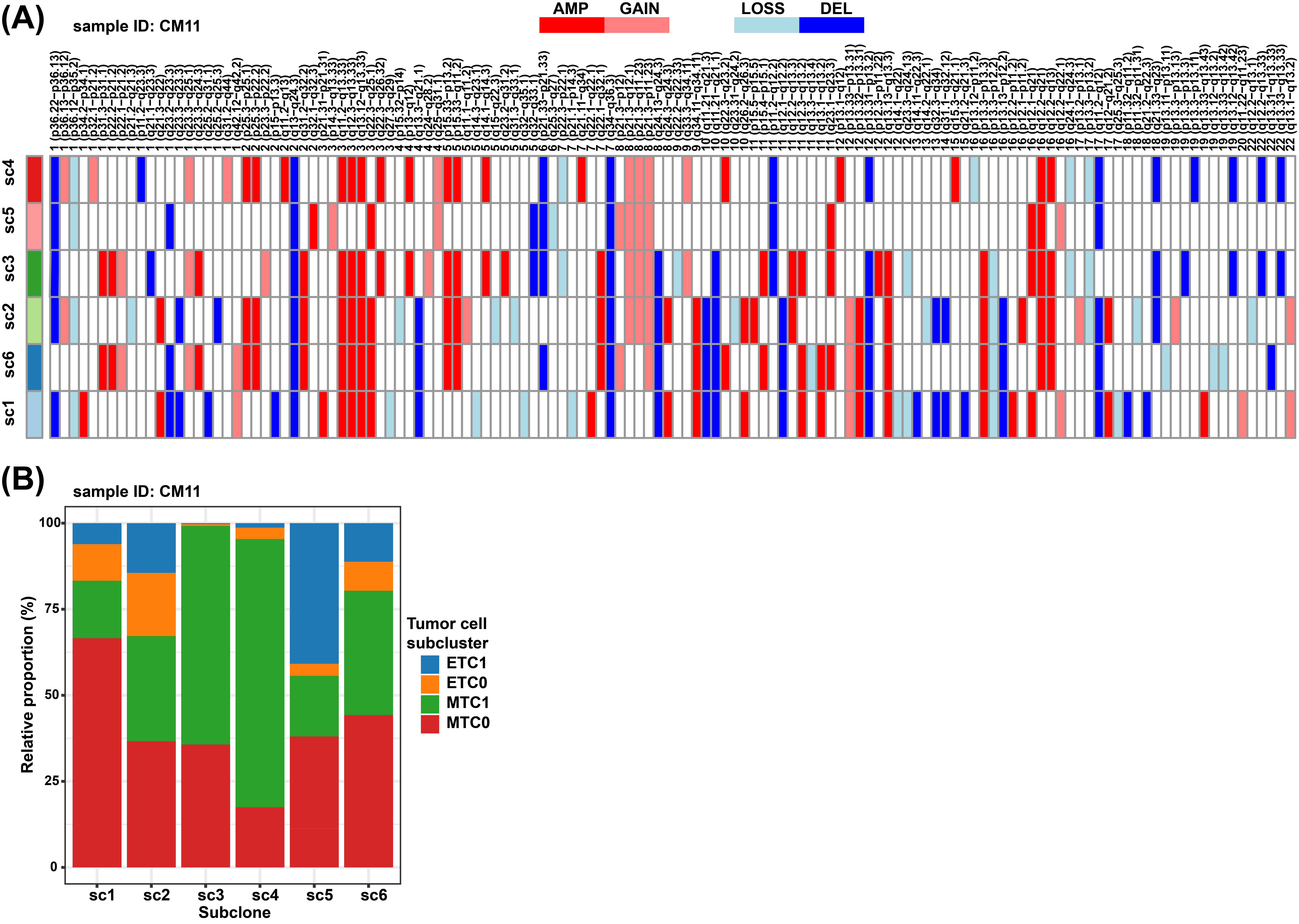
Figure S**2 Intratumoral heterogeneity of myxoma tumor cells in sample CM11. (A)** OncoPrint-like plot showing CNVs shared by subclones and subclone-specific CNVs. **(B)** Relative proportion of each tumor cell subcluster in each subclone.

**
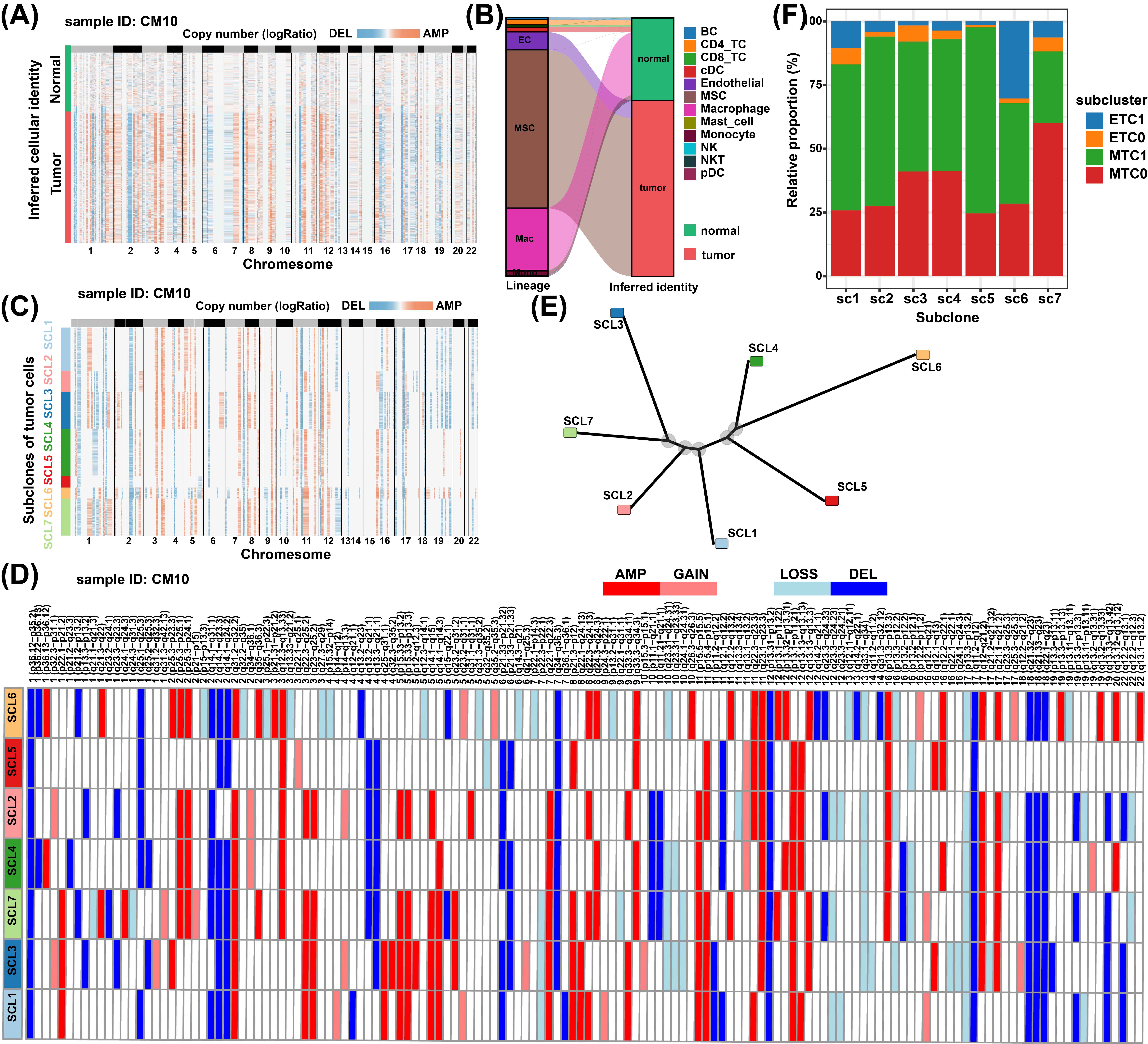
**

## Figure S**3 Normal/tumor cell classification and clonal substructure inference of cardiac myxoma in sample CM10. (A)** Classification of myxoma cells as tumor cells and normal cells based on copy number profile in sample CM10. **(B)** Alluvial plot showing that the myoma cells with EC or MSC identities were classified as tumor cells in sample CM10. **(C)** Copy number profile of each subclone in tumor cells of sample CM10. **(D)** OncoPrint-like plot showing CNVs shared by subclones and subclone-specific CNVs. **(E)** Phylogenetic tree showing intratumoral clonal substructure of sample CM10. **(F)** Relative proportion of each tumor cell subcluster in each subclone.

**
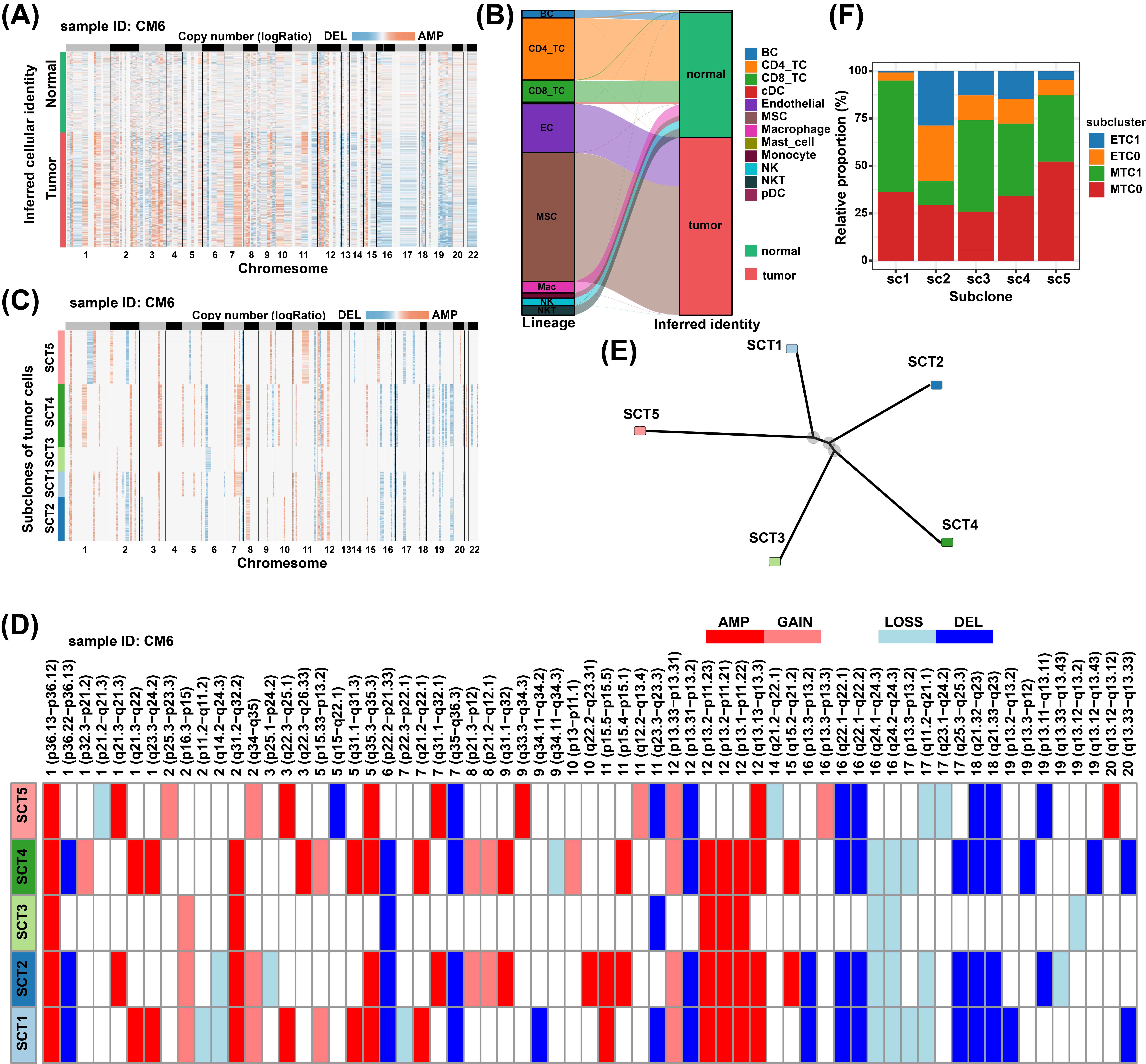
**

## Figure S**4 Normal/tumor cell classification and clonal substructure inference of cardiac myxoma in sample CM6. (A)** Classification of myxoma cells as tumor cells and normal cells based on copy number profile in sample CM6. **(B)** Alluvial plot showing that the myoma cells with EC or MSC identities were classified as tumor cells in sample CM6. **(C)** Copy number profile of each subclone in tumor cells of sample CM6. **(D)** OncoPrint-like plot showing CNVs shared by subclones and subclone-specific CNVs. **(E)** Phylogenetic tree showing intratumoral clonal substructure of sample CM6. **(F)** Relative proportion of each tumor cell subcluster in each subclone.

##
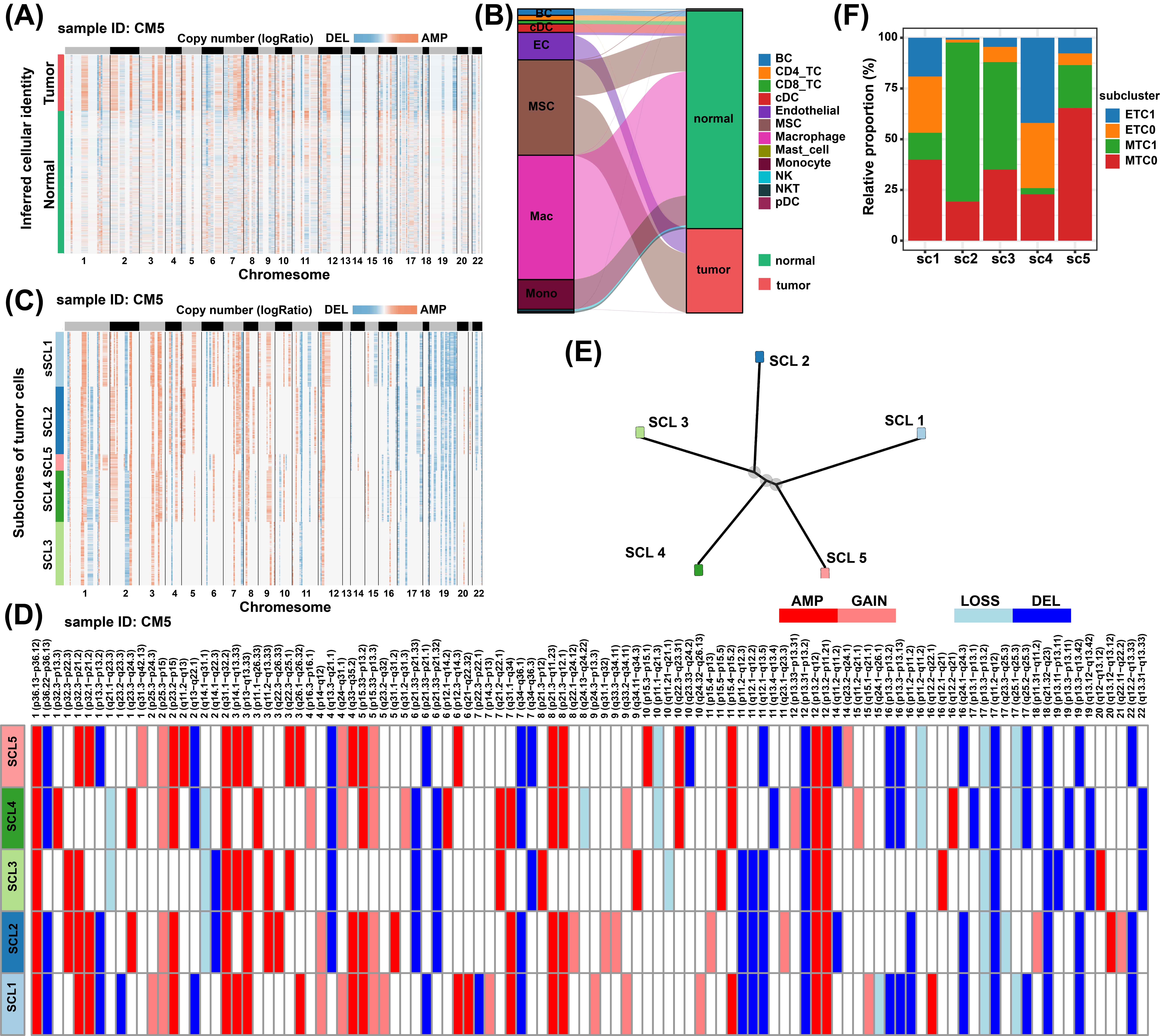
Figure S**5 Normal/tumor cell classification and clonal substructure inference of cardiac myxoma in sample CM5. (A)** Classification of myxoma cells as tumor cells and normal cells based on copy number profile in sample CM5. **(B)** Alluvial plot showing that the myoma cells with EC or MSC identities were classified as tumor cells in sample CM5. **(C)** Copy number profile of each subclone in tumor cells of sample CM5. **(D)** OncoPrint-like plot showing CNVs shared by subclones and subclone-specific CNVs. **(E)** Phylogenetic tree showing intratumoral clonal substructure of sample CM5. **(F)** Relative proportion of each tumor cell subcluster in each subclone.

##
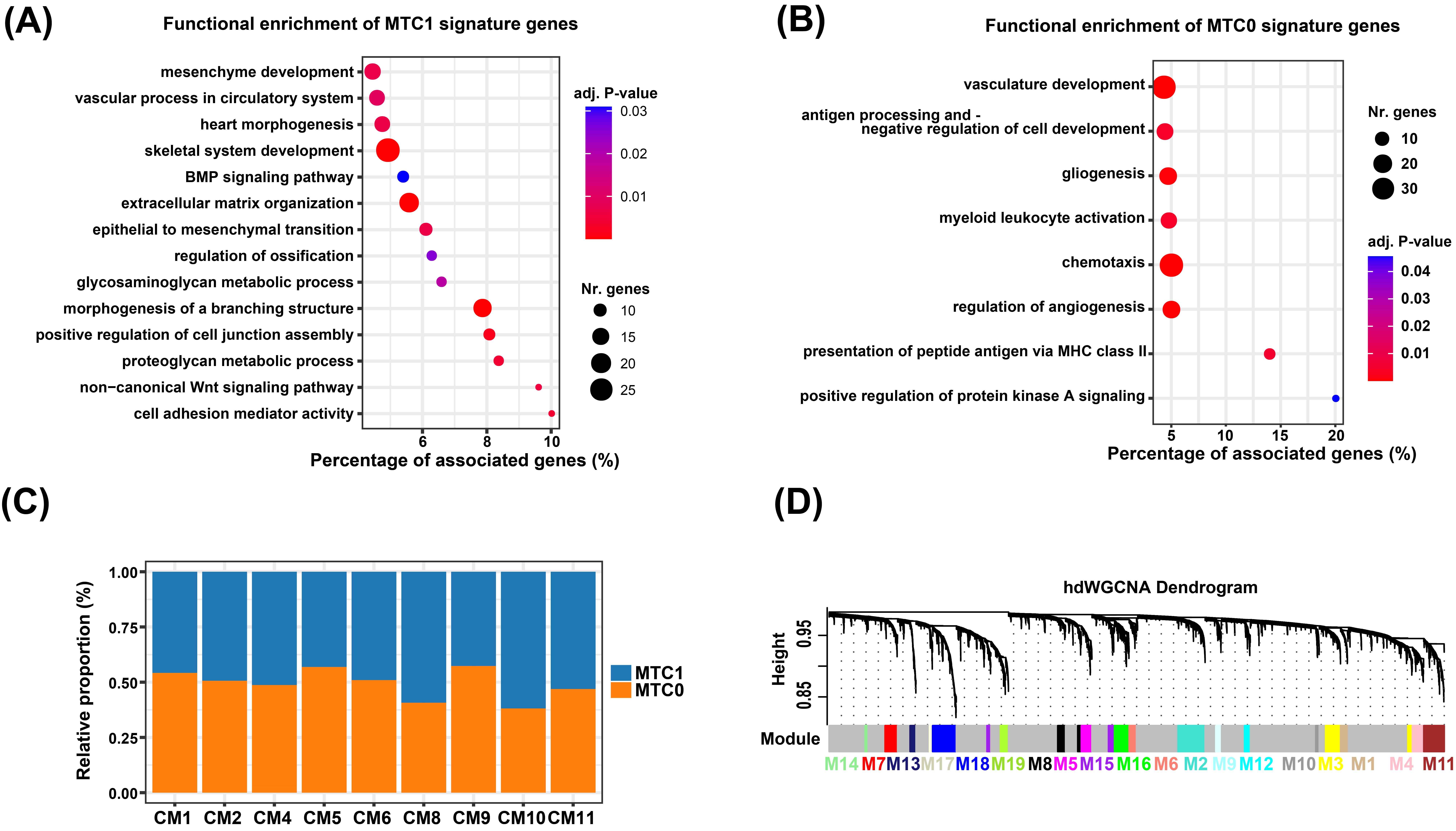
Figure S6 Functional enrichment and relative proportion of the subclusters of MSC-like tumor cells in cardiac myxoma. (A) Function enrichment of the signature genes of subcluster MTC0. (B) Function enrichment of the signature genes of subcluster MTC1. (C) Relative proportion of subclusters in each sample. In A and B, only representative terms of Gene Ontology biological processes are shown. Hypergeometric tests were performed for the functional enrichment analysis using ClueGO (significance threshold: P-value adjusted for multiple testing < 0.05). (D) Dendrogram showing gene co-expression modules identified by co-expression network analysis using hdWGCNA.

##
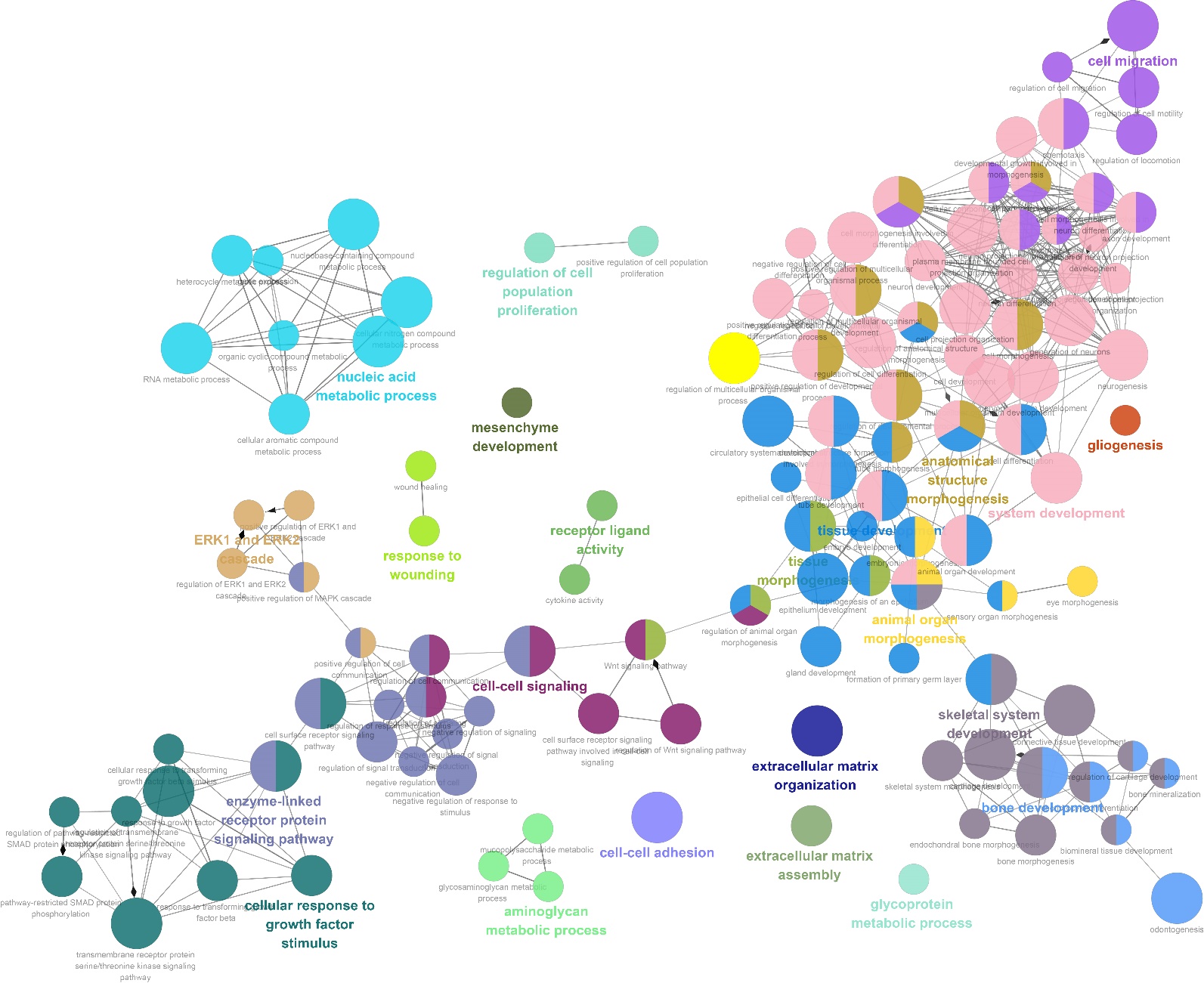
Figure S**7** **Functional enrichment of the** differentially expressed genes between EC-like tumor cells in cardiac myxoma and left atrial ECs in normal hearts. The expression data of normal hearts were downloaded from the Heart Cell Atlas database (https://www.heartcellatlas.org/v1.html).



## **Figure S8 Functional enrichment, regulon activity, co-expression network, and relative proportion of the subclusters of EC-like tumor cells in cardiac myxoma. (A)** Expression of markers for the endothelial lineage in each subcluster. **(B)** Function enrichment of the signature genes of subcluster ETC0. **(C)** Function enrichment of the signature genes of subcluster ETC1. (D) Dendrogram showing gene co-expression modules identified by co-expression network analysis using hdWGCNA. **(E)** Gene co-expression network of EC-like tumor cells. Each dot denotes a single gene. The dot is color-coded by gene module. The dot size is scaled by the gene’s eigengene-based connectivity (kME). The top three genes ranked by kME of each module are shown. **(F)** Relative proportion of subclusters in each sample. In B and C, only representative terms of Gene Ontology biological processes are shown. Hypergeometric tests were performed for the functional enrichment analysis using ClueGO (significance threshold: P-value adjusted for multiple testing < 0.05).

##
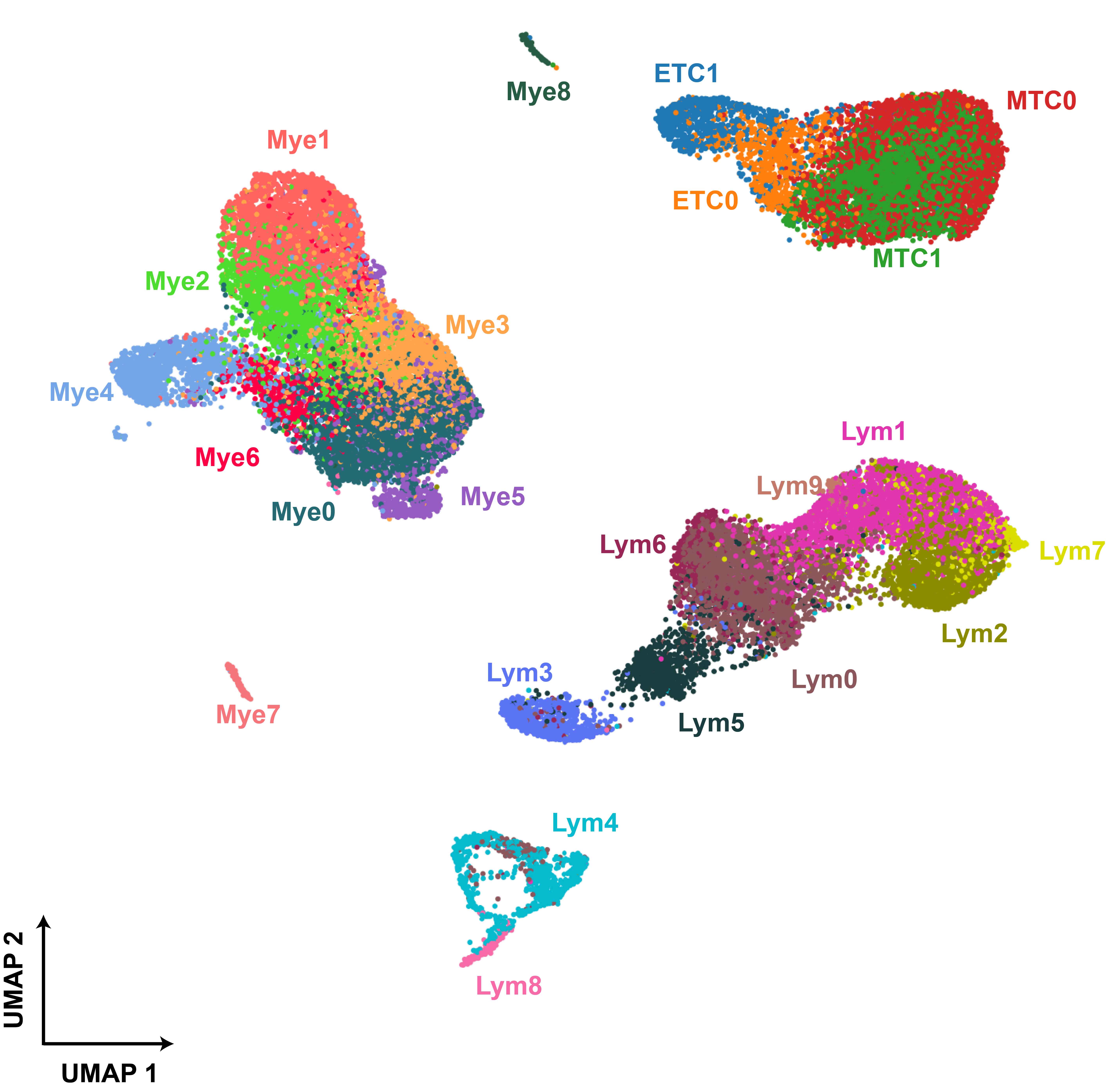
Figure S9 UMAP plots showing the distribution of cellular subclusters.

##
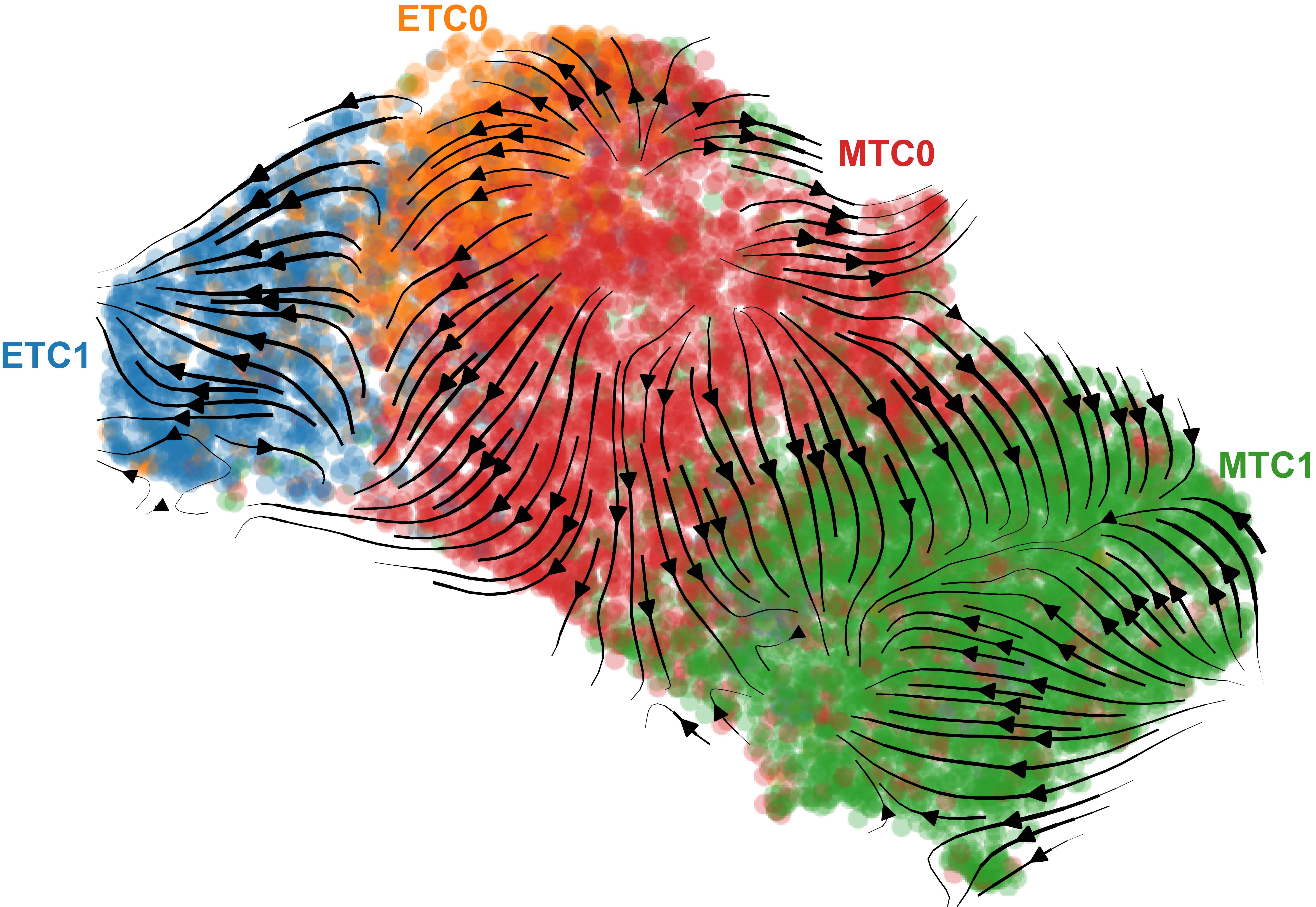
Figure S10 RNA velocity analysis of all myxoma tumor cells (MTCs and ETCs).

##
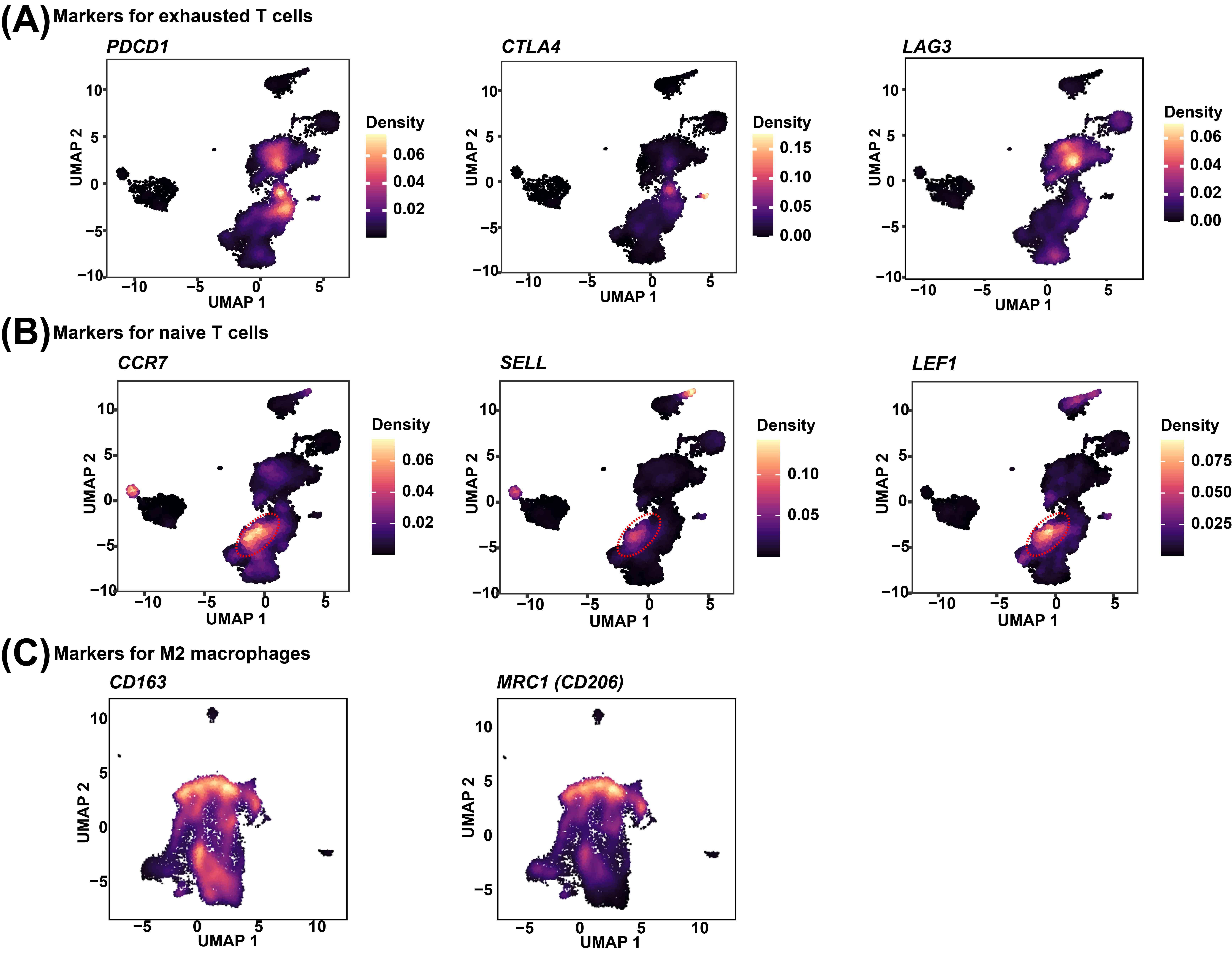
Figure S**11 UMAP plots showing expression of markers for exhausted or naive T cells in lymphoid cells and markers for M2 macrophages in myeloid cells. (A)** Expression of markers for exhausted T cells in lymphoid cells. **(B)** Expression of markers for naive T cells in lymphoid cells. **(C)** Expression of markers for M2 macrophages in myeloid cells. The visualization was enhanced by gene-weighted density estimation using the R package Nebulosa to recover the signal from dropped-out features.


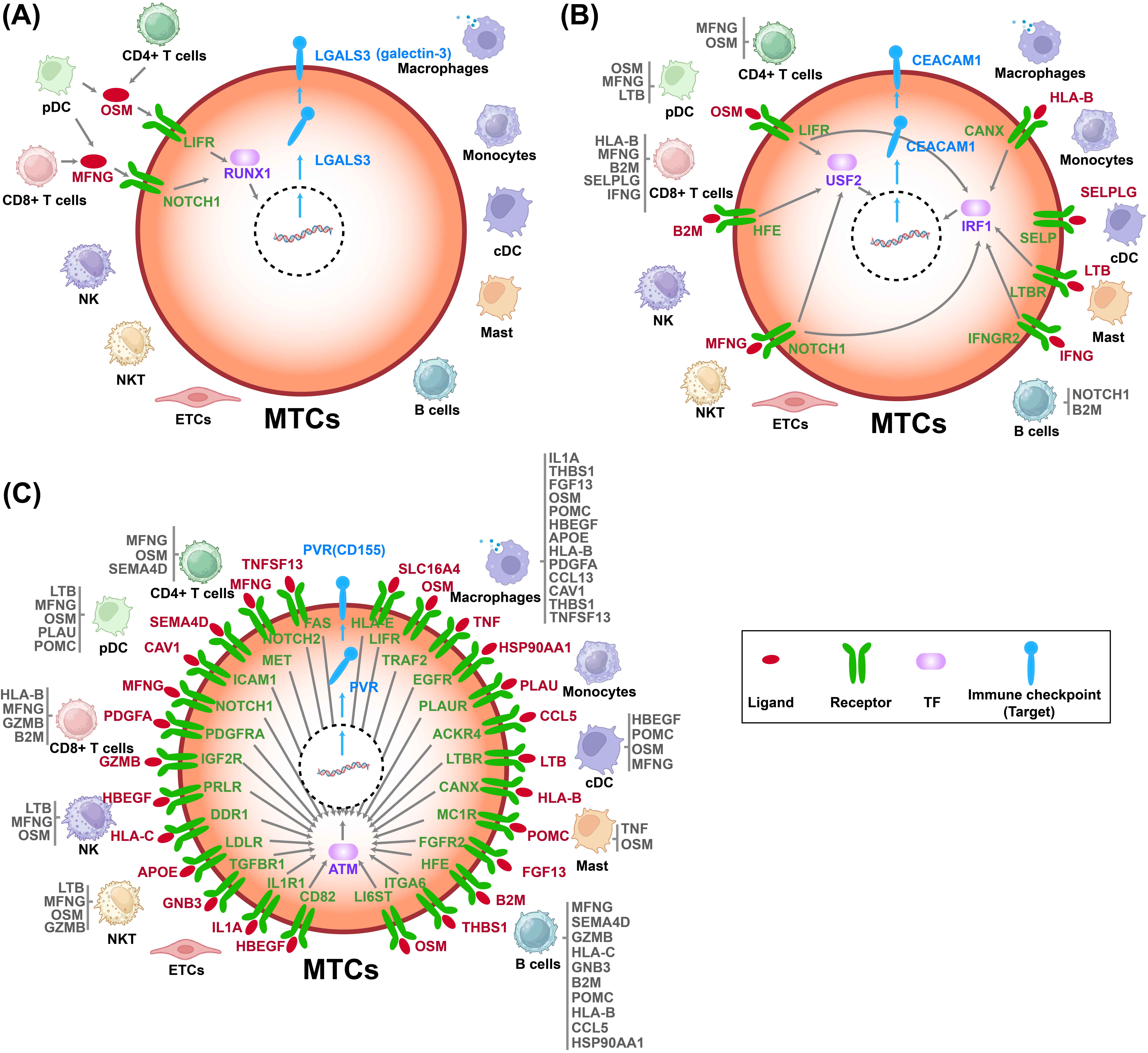


## Figure S12 Intracellular regulatory networks governing the expression of inhibitory immune checkpoint ligands in MTCs activated potentially through intercellular communications. (A) Regulation of the expression of LGALS3 (galectin-3) in MTCs. (B) Regulation of the expression of CEACAM1 in MTCs. (C) Regulation of the expression of PVR (CD155) in MTCs. A multilayer regulatory network, encompassing ligand-receptor, receptor-TF, and TF-target gene subnetworks, was deduced based on the scRNA-seq data using scMLnet. For other inhibitory immune checkpoints expressed in CM tumor cells, such as (CD274) PD-L1, IGSF11 (VSIG-3), and NECTIN2 (CD112), scMLnet was unable to deduce an intracellular regulatory network. cDC: conventional plasmacytoid dendritic cell, ETC: EC-like tumor cell, MTC: MSC-like tumor cell. NK: natural killer cell, NKT: natural killer T cell, pDC: plasmacytoid dendritic cell, TC: T cell, TF: Transcription factor. In B and C, gene symbols listed in gray next to a cell type represent potential ligands expressed by that particular cell type.





## Figure S13 H&E staining and ST spot cluster distribution on three sections of cardiac myxoma. (A) H&E staining (left) and ST spot cluster distribution (right) for a section of sample S84444. (B) H&E staining (left) and ST spot cluster distribution (right) for a section of sample S81631. (C) H&E staining (left) and ST spot cluster distribution (right) for a section of sample S73042. (D) H&E staining (left) and ST spot cluster distribution (right) for a section of sample S82094. Spots are color-coded by spot cluster. Scale bar: 1 mm.


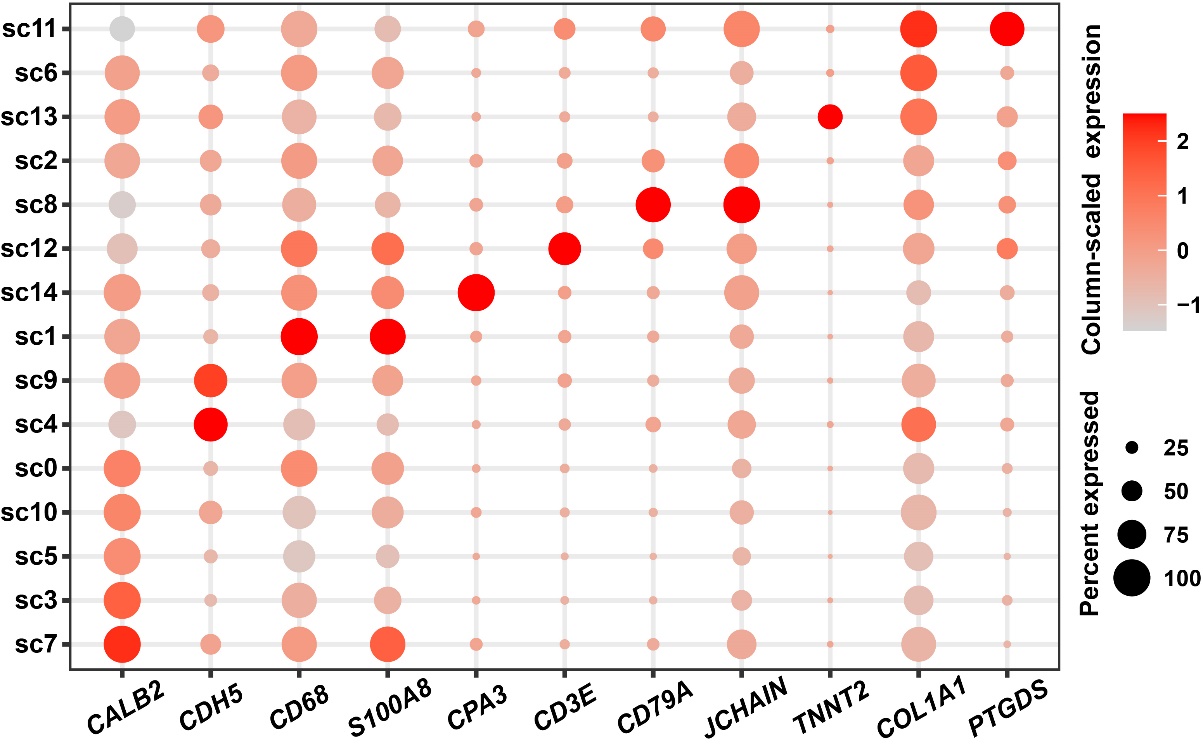


## Figure S14 Expression of representative marker genes in each spot cluster.





## Figure S15 Predicted composition of cell types (A) and subclusters (B) at the spot level for the CM tissue section S81555. The pie chart on the right shows the overall composition of the section. Potential noise labels were filtered based on quantile (threshold=0.5) using the tool stLearn. The prediction was conducted through the integration of snRNA-seq and ST data using Seurat's label transfer workflow.





## Figure S16 Predicted composition of cell types (A) and subclusters (B) at the spot level for the CM tissue section S84444. The pie chart on the right shows the overall composition of the section. Potential noise labels were filtered based on quantile (threshold=0.5) using the tool stLearn. The prediction was conducted through the integration of snRNA-seq and ST data using Seurat's label transfer workflow.


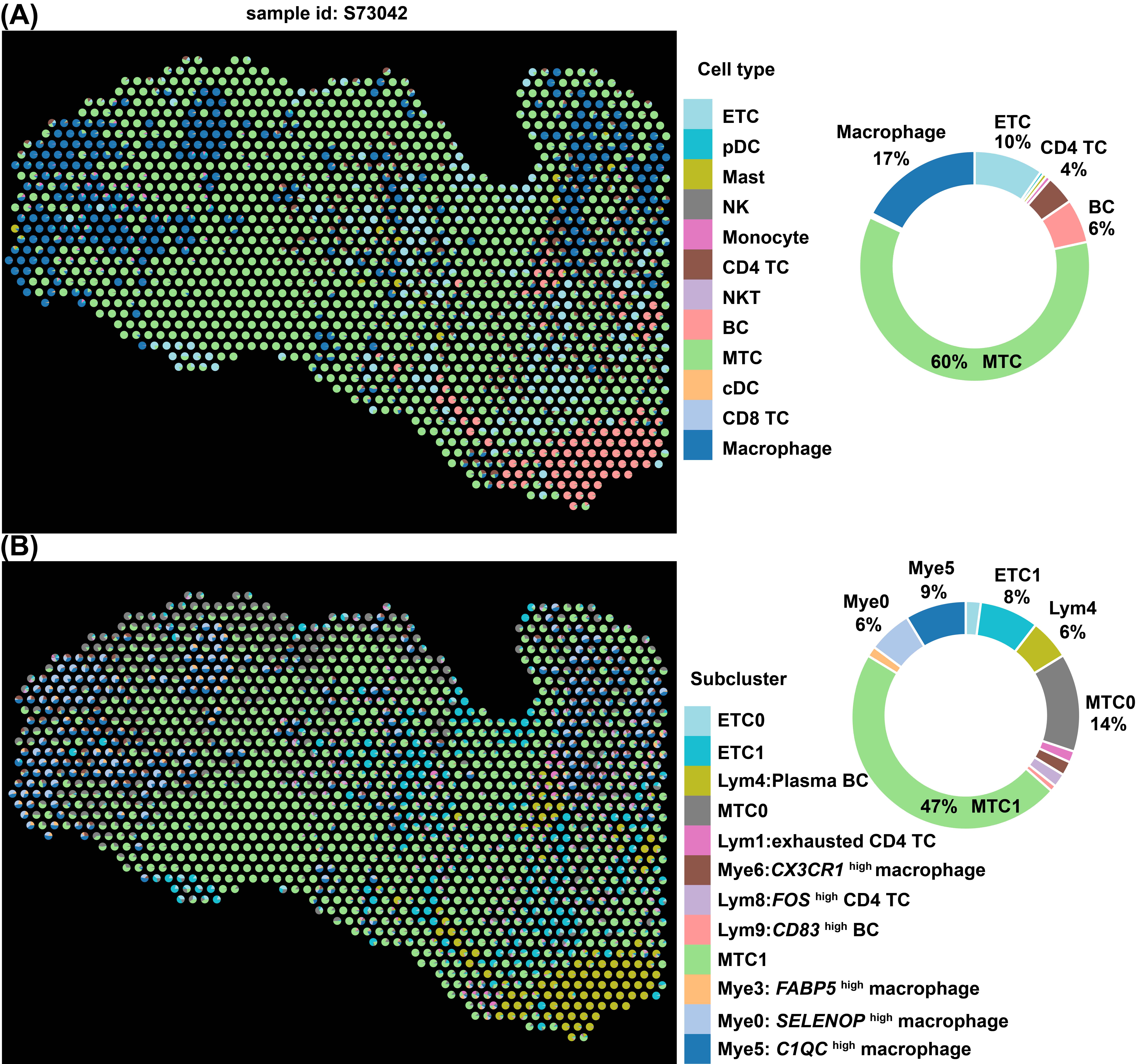


## Figure S17 Predicted composition of cell types (A) and subclusters (B) at the spot level for the CM tissue section S73042. The pie chart on the right shows the overall composition of the section. Potential noise labels were filtered based on quantile (threshold=0.5) using the tool stLearn. The prediction was conducted through the integration of snRNA-seq and ST data using Seurat's label transfer workflow.





## Figure S18 Predicted composition of cell types (A) and subclusters (B) at the spot level for the CM tissue section S81631. The pie chart on the right shows the overall composition of the section. Potential noise labels were filtered based on quantile (threshold=0.5) using the tool stLearn. The prediction was conducted through the integration of snRNA-seq and ST data using Seurat's label transfer workflow.





## Figure S19 Predicted composition of cell types (A) and subclusters (B) at the spot level for the CM tissue section S82094. The pie chart on the right shows the overall composition of the section. Potential noise labels were filtered based on quantile (threshold=0.5) using the tool stLearn. The prediction was conducted through the integration of snRNA-seq and ST data using Seurat's label transfer workflow.





## Figure S20 Spatially proximal cell-cell communications inferred from spatial transcriptomic data. (A) The predicted dominant cell type for each ST spot of the section S81555. The prediction was conducted through the integration of snRNA-seq and ST data using Seurat's label transfer workflow. (B) Heatmap showing interaction strength among cell types. The bar denotes the accumulated strength of interactions that were sent (right) or received (top) by each cell type. (C) Heatmap showing the number of interactions among cell types. The bar denotes the total number of interactions that were sent (right) or received (top) by each cell type. (D) Strength of secreted signaling pathways sent (outgoing) or received (incoming) by each cell type. The top bars denote the accumulated strength of signals that were sent or received by each cell type. Right bars denote the accumulated strength of signals across all cell types. (E) Inferred network for CHEMERIN signaling pathway (left) and binary expression pattern of the ligand-receptor pair RARRES2-CMKLR1 (right). (F) Inferred network for HGF signaling pathway (left) and binary expression pattern of the ligand-receptor pair HGF-MET (right). (G) Inferred network for GDF signaling pathway (left) and binary expression pattern of the ligand-receptor pair GDF15-TGFBR2 (right). In E-G, each dot in the network represents a specific cell type, and the size of the dot is weighted according to the strength of the outgoing signaling from that cell type. The width of the edge is weighted by the strength of intercellular communication.
